# Mechanistic Profiling of Phenethyl Isothiocyanate in CCRF-CEM Acute Lymphoblastic Leukemia Cells

**DOI:** 10.3390/ph19101631

**Published:** 2026-10-14

**Authors:** Shila Prajapati, Xiao Lei, Mohamed Elbadawi, Mona Dawood, Sabine M. Klauck, Wan Gong, Letian Shan, Percy Kampeis, Thomas Efferth, Xiaohua Lu

**Affiliations:** 1 Department of Pharmaceutical Biology, Institute of Pharmaceutical and Biomedical Sciences, Johannes Gutenberg University Mainz, 55128 Mainz, Germany; 2 Department of Molecular Biology, Faculty of Medical Laboratory Science, Al-Neelain University, Khartoum 12702, Sudan; 3 Division of Cancer Genome Research, German Cancer Research Center (DKFZ), Im Neuenheimer Feld 280, 69120 Heidelberg, Germany; 4 National Center for Tumor Diseases (NCT) Heidelberg, Im Neuenheimer Feld 460, 69120 Heidelberg, Germany; 5 Fuyang Institute, Zhejiang Chinese Medical University, Hangzhou 311422, China; 6 Institute for Biotechnical Process Design, Trier University of Applied Sciences, Environmental Campus Birkenfeld, Campusallee 9913, 55768 Hoppstädten-Weiersbach, Germany

**Keywords:** cancer, isothiocyanates, network pharmacology, microtubules, zebrafish xenograft

## Abstract

**Background/Objectives:** Phenethyl isothiocyanate (PEITC), a bioactive compound found in cruciferous vegetables, exhibits potent anti-cancer activity, but its molecular mechanisms in acute lymphoblastic leukemia (ALL) remain poorly defined. This study aimed to investigate the anti-leukemic effects of PEITC. **Methods:** PEITC cytotoxicity was assessed in human cancer cell lines using resazurin reduction assays. Microarray and Ingenuity Pathway Analysis (IPA) were used to investigate altered molecular signaling pathways. Functional assays were performed to assess DNA damage, cell-cycle progression, apoptosis, autophagy, mitochondrial membrane potential, microtubule organization and endothelial tube formation. Western blotting was performed to examine pathway-related proteins, and preliminary in vivo antitumor activity was evaluated using a CCRF-CEM zebrafish xenograft model. **Results:** PEITC exerted potent cytotoxicity against CCRF-CEM cells (IC_50_ = 1.76 ± 0.07 µM). Microarray and IPA analyses predicted inhibition of PI3K/AKT/mTOR signaling, which was further supported by Western blot analysis. Functional assays demonstrated that PEITC induced DNA damage, cell cycle perturbation, mitochondrial dysfunction, and apoptotic and autophagic responses. PEITC also disrupted microtubule organization in GFP-tagged α-tubulin-expressing U2OS cells. Furthermore, PEITC suppressed endothelial tube formation in EA.hy926 cell lines and exhibited preliminary in vivo antitumor activity in a zebrafish xenograft model (*n* = 10). **Conclusions:** These findings provide mechanistic insights into the anti-leukemic activity of PEITC and demonstrate its effects on multiple cellular processes associated with leukemia cell survival and proliferation, supporting further investigation of its therapeutic potential in ALL.

## 1. Introduction

Natural products have attracted significant attention over the past decades for their promising role in anticancer research. Among them, dietary isothiocyanates (ITCs), bioactive phytochemicals abundant in cruciferous vegetables such as broccoli, cabbage, cauliflower, Brussels sprouts, and mustard, have been widely studied for their chemopreventive properties [1]. Epidemiological evidence has demonstrated that the consumption of cruciferous vegetables is associated with reduced risk of cancer [2]. ITCs are derived from glucosinolates through hydrolysis catalysed by the enzyme myrosinase upon disruption of plant tissue, forming a core component of the plant’s defence mechanism against environmental stress, climate change, or pathogens [3]. The anticancer activity of ITCs is largely attributed to the presence of the -N=C=S functional group, which is crucial to their biological activity [4].

Phenethyl isothiocyanate (PEITC), one of the constituents of ITCs, exhibits diverse biological effects, including antimicrobial [5], anti-inflammatory [6], anti-angiogenic [7], and anticancer activities [8]. The cytotoxic effects of PEITC have been demonstrated in both human cancer cell lines and animal models. Specifically, PEITC inhibits the proliferation of cervical cancer [9], ovarian cancer [10], and brain glioblastoma [11] by inducing caspase-dependent apoptotic cell death. Moreover, PEITC suppresses cellular invasion and migration in hepatocellular carcinoma [12], prostate cancer [13], lung cancer [14], colorectal cancer [15], and melanoma [16]. PEITC has also been reported to induce DNA damage and modulate DNA repair pathways in human retinoblastoma cells [17]. In pre-clinical settings, PEITC has demonstrated antitumor activity in various tumor xenograft models, including breast cancer [18] and glioblastoma [19], and has been shown to enhance the sensitivity of non-small cell lung cancer (NSCLC) cells to cisplatin by targeting c-Myc and modulating the PI3K/AKT/mTOR signaling pathway [20].

Acute lymphoblastic leukemia (ALL), a rare disease with an estimated 6250 new cases diagnosed and 1300 deaths from ALL in the United States in 2026, arises from the malignant transformation of immature lymphocytes and is frequently associated with significant chromosomal abnormalities [21]. These molecular aberrations contribute to the dysregulation of key signaling pathways, such as NOTCH, MAPK, and PI3K/AKT/mTOR, which play critical roles in leukemogenesis and disease progression [22]. The PI3K/AKT/mTOR signaling axis serves as a central regulator of cellular growth, survival, and stress responses. Beyond its role in promoting cell proliferation, this pathway modulates multiple proteins involved in DNA damage response and repair, contributing to genomic integrity [23]. This pathway also regulates cytoskeletal organization and microtubule dynamics during cell division, while mTOR acts as a key negative regulator of autophagy initiation [24]. Aberrant activation of the PI3K/AKT/mTOR pathway enhances cancer cell survival and resistance to apoptosis, thereby promoting the persistence of cells with genomic abnormalities and contributing to disease progression [25].

In ALL pathogenesis, the loss or inactivation of PTEN activates the PI3K/AKT/mTOR pathway, thereby promoting increased cell proliferation and chemoresistance [26]. Hence, targeting this signaling axis has emerged as an important therapeutic strategy for haematological malignancies. Indeed, combination therapies involving dual PI3K/mTOR inhibitors or mTOR inhibitors used in conjunction with other pathway-targeting agents have shown promising therapeutic potential in leukaemia treatment [27]. With deeper insight into the biology of ALL, novel therapies spanning intensive chemotherapy, immunotherapy, and targeted treatment continue to be pursued [28]. However, disease relapse occurs frequently within the first two years and carries a poor prognosis. These clinical challenges underscore the urgent need for novel multitarget therapeutic approaches to improve treatment outcomes.

Despite extensive research on the therapeutic potential of PEITC in solid cancer types, its activity and underlying molecular mechanisms in ALL remain insufficiently characterized. It is still unclear how PEITC affects signaling pathways relevant to ALL cell survival and whether these effects are associated with cellular responses such as DNA damage, cell cycle arrest, and cell death. In the present study, we investigated the cytotoxic effects of PEITC across a panel of cancer cell lines, with human T-cell acute lymphoblastic leukemia CCRF-CEM cells exhibiting the highest sensitivity. Using an integrated approach, we evaluated the transcriptomics-based signaling networks in CCRF-CEM cells, focusing on how PEITC treatment alters the PI3K/AKT/mTOR axis. We then phenotypically validated these network predictions through an independent suite of biological assays assessing upstream genomic and structural stress, specifically DNA damage, cell cycle progression, and microtubule network integrity, as well as downstream survival pathways, including apoptosis and autophagy.

## 2. Results

### 2.1. PEITC Exhibited Cytotoxicity in Cancer Cell Lines

**Figure 1:**
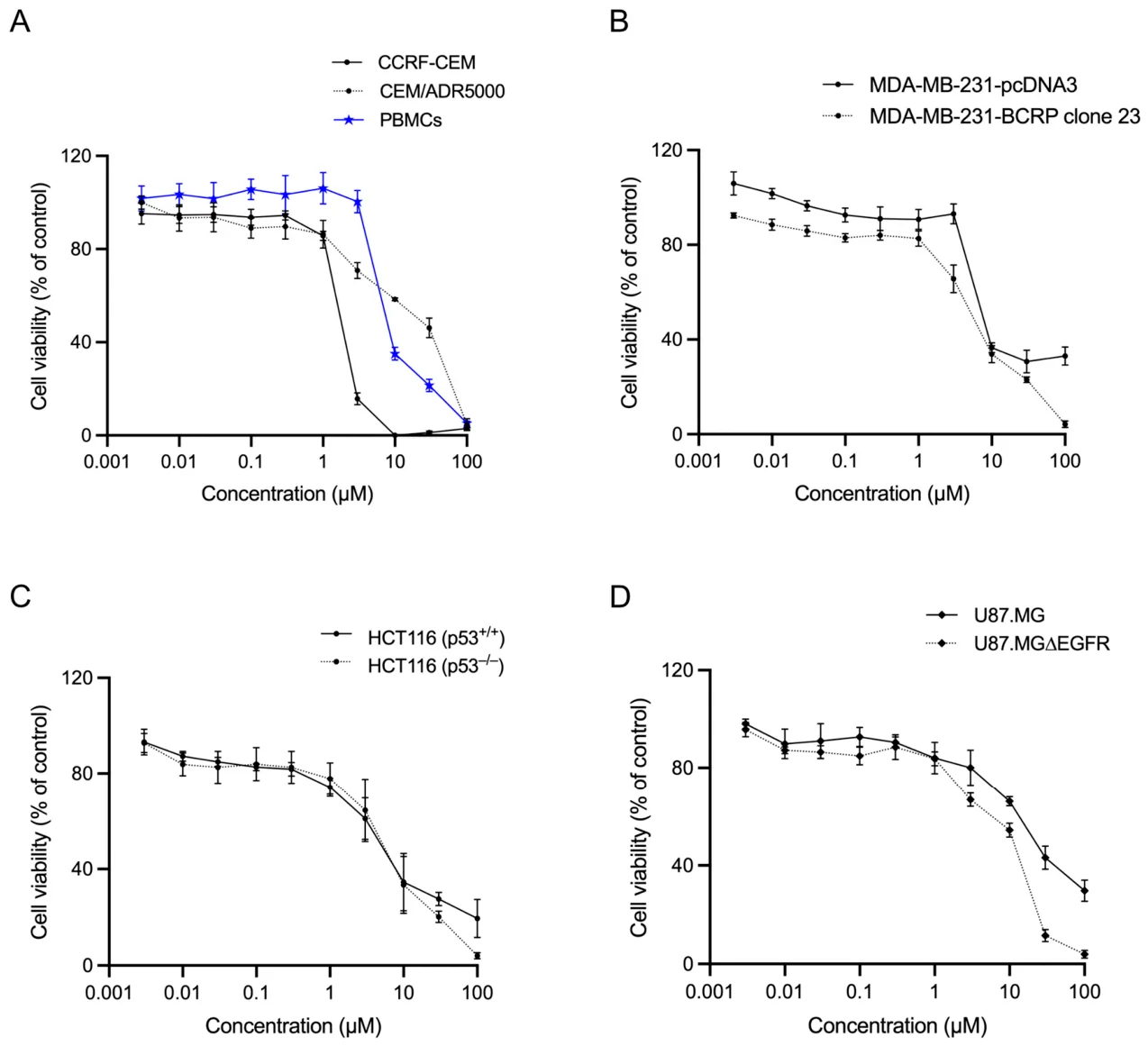
Dose–response curves of PEITC as determined by the resazurin assay. Effect of PEITC on the cell viability of (**A**) CCRF-CEM, *P*-gp-overexpressing CEM/ADR5000 leukemia cell lines and human peripheral blood mononuclear cells (PBMCs); (**B**) MDA-MB-231-pcDNA3 and its transduced MDA-MB-231-BCRP clone 23 breast cancer cells; (**C**) HCT116 (p53^+/+^) and p53^−/−^ knockout HCT116 (p53^−/−^) colon cancer cell lines; and (**D**) U87.MG wild-type human glioblastoma cells and transfected U87.MGΔEGFR cells. The x-axis represents PEITC concentration (µM), and the y-axis represents cell viability (%). Data are presented as mean ± SD from three independent biological experiments (*n* = 3), with six technical replicates per concentration in each experiment.

A panel of cancer cell lines was included to evaluate the cytotoxic activity of PEITC across different cancer models, including cell lines with distinct drug-resistance characteristics, such as *P*-glycoprotein (*P*-gp) and breast cancer resistance protein (BCRP) overexpression, as well as different p53 and EGFR statuses (Figure 1A–D). After 72 h of exposure, PEITC showed greater cytotoxicity toward drug-sensitive CCRF-CEM cells (IC_50_ = 1.76 ± 0.07 µM) compared to the *P*-gp-overexpressing CEM/ADR5000 (IC_50_ = 22.98 ± 6.82 µM). In contrast, BCRP-overexpressing MDA-MB-231-BCRP clone 23 cells and U87.MGΔEGFR cells were more sensitive to PEITC than their parental cells, with resistance ratios of 0.71 and 0.49, respectively. PEITC demonstrated similar cytotoxicity towards HCT116 p53^+/+^ wild-type and HCT116 p53^−/−^ knockout cells with IC_50_ values of 5.63 ± 3.04 and 5.66 ± 2.88 µM, respectively. Meanwhile, PBMCs were less sensitive to PEITC (IC_50_ = 7.61 ± 0.26 µM) than CCRF-CEM cells, with an approximately fourfold higher IC_50_ value (Table 1). Among the tested cancer cell models, CCRF-CEM cells were the most sensitive to PEITC and were therefore selected for subsequent mechanistic studies.

### 2.2. Microarray Hybridization-Based Expression Profiling and qRT-PCR

**Figure 2:**
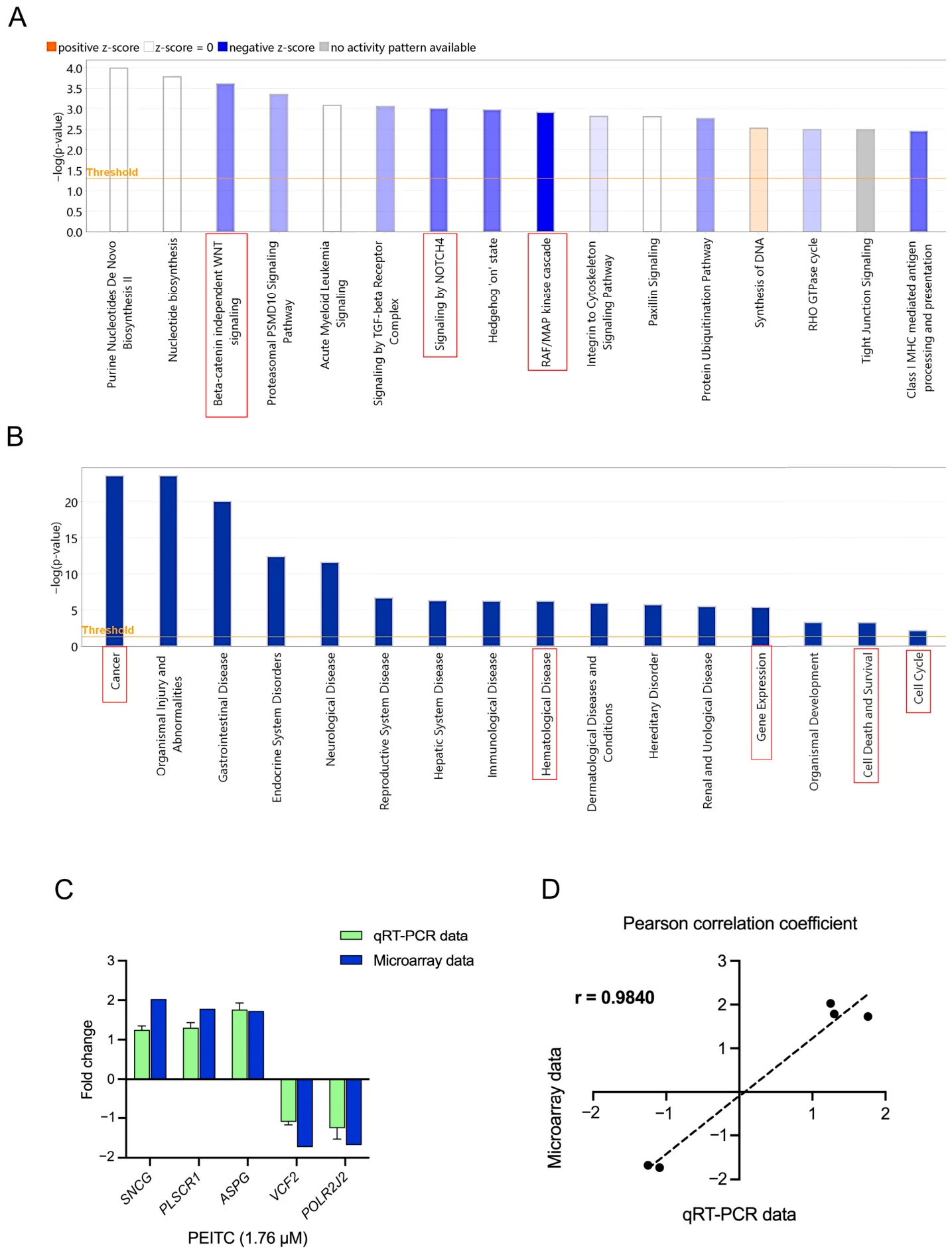
Microarray gene expression profiling and IPA of CCRF-CEM cells following PEITC treatment. CCRF-CEM cells were treated with PEITC or with DMSO vehicle control for 24 h, followed by microarray-based gene-expression profiling. (**A**) Canonical pathways affected by PEITC. (**B**) Top cellular functions and diseases identified by IPA based on differentially expressed genes. Important signaling pathways and cellular functions are highlighted in red rectangles. The x-axis represents the −log(*p*-value), where longer bars indicate higher significance. The yellow line indicates the significance threshold (log(*p*-value) = 1.3). (**C**) Technical verification of microarray-based gene expression of five selected genes (*SNCG*, *PLSCR1*, *ASPG*, *VCF2*, and *POLR2J2*) by qRT-PCR. (**D**) Correlation between gene-expression changes determined by microarray analysis and qRT-PCR. Pearson correlation analysis showed a strong positive correlation between the two datasets (*r* = 0.984).

To investigate the molecular mechanisms associated with the response of CCRF-CEM cells to PEITC, microarray-based expression profiling was performed (*n* = 2 independent biological experiments). Differentially expressed genes were identified by comparing PEITC-treated and DMSO control cells using an empirical Bayes moderated *t*-test with Benjamini–Hochberg correction (false discovery rate (FDR)-adjusted *p* < 0.05) and subsequently analyzed using Ingenuity Pathway Analysis (IPA). IPA identified canonical pathways associated with the PEITC-induced gene-expression profile, including “WNT signaling”, “NOTCH4 signaling”, and “RAF/MAP kinase” (Figure 2A). IPA further identified “Cancer”, “Gene Expression”, “Haematological Disease”, “Cell Death and Survival”, and “Cell Cycle”, among the major diseases and cellular functions associated with the differentially expressed genes (Figure 2B). A total of 218 genes were significantly differentially expressed between PEITC-treated and DMSO control cells, comprising 95 upregulated and 123 downregulated genes (Supplementary Table S1). Among the differentially expressed genes, *SNCG*, *PLSCR1* and *ASPG* were upregulated by 2.03-, 1.78- and 1.72-fold, respectively, whereas *VCF2* and *POLR2J2* showed fold changes of -1.73 and -1.68, respectively. These top upregulated and downregulated genes were selected for technical verification by qRT-PCR. The expression patterns observed by quantitative reverse transcription polymerase chain reaction (qRT-PCR) were consistent with the microarray data, demonstrating upregulation of *SNCG*, *PLSCR1*, and *ASPG* and downregulation of *VCF2* and *POLR2J2*, as supported by a strong Pearson correlation coefficient (*r* = 0.984) (Figure 2C,D).

### 2.3. PEITC Supressed PI3K/AKT/mTOR/NF-κB-Associated Signaling Pathways in CCRF-CEM Cells

**Figure 3:**
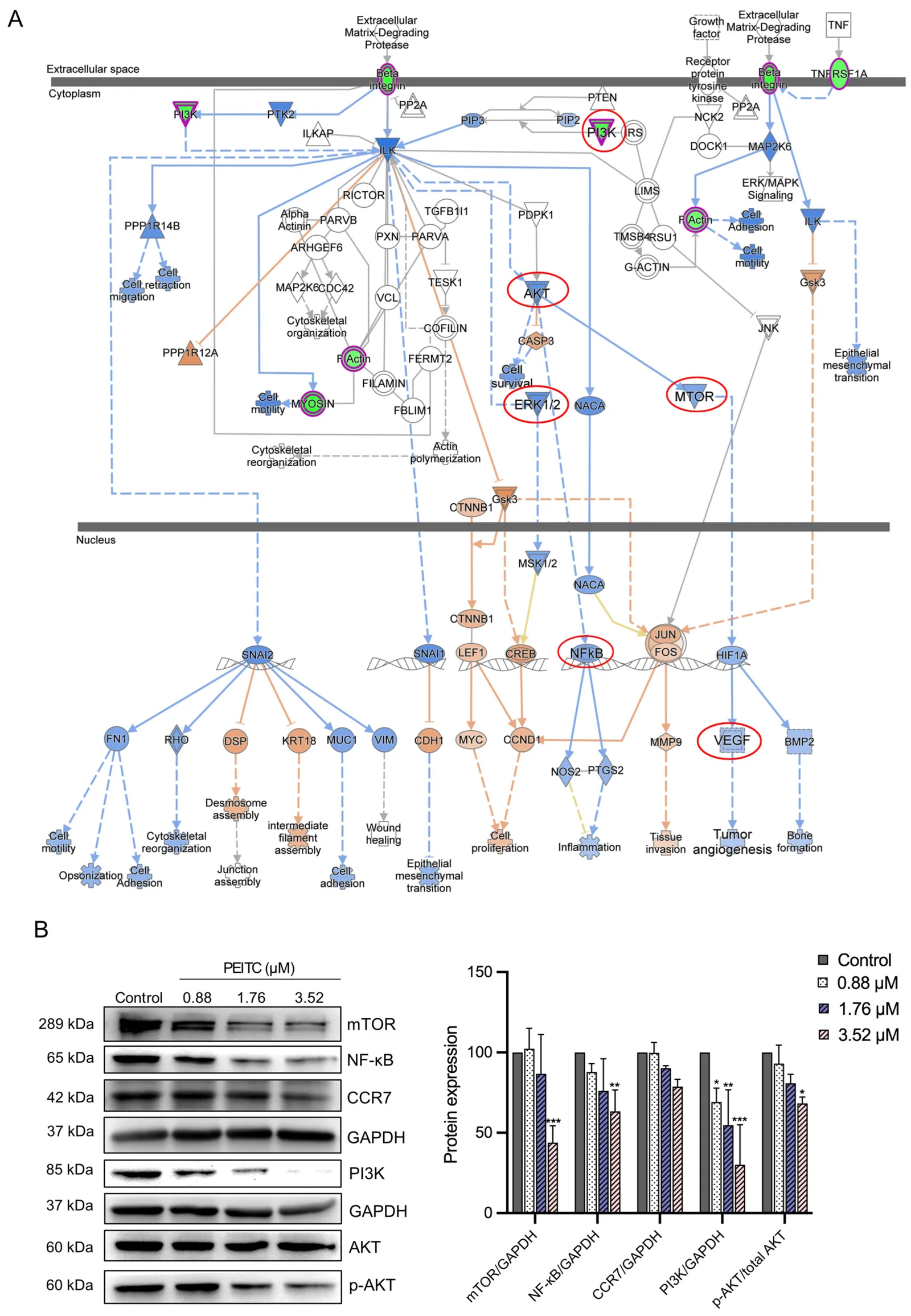
IPA-predicted signaling changes and protein expressions associated with PEITC treatment in CCRF-CEM cells. (**A**) IPA analysis of the PEITC-associated gene expression profile predicted alterations in cancer-related signaling pathways, including PI3K/AKT/mTOR signaling, as well as biological functions related to cell survival and tumor angiogenesis, highlighted by red circles. In the IPA network, blue and green indicate predicted inhibition or downregulation, whereas red and orange indicate predicted activation or upregulation, respectively. The solid lines indicate direct molecular relationships, whereas dashed lines represent indirect relationships between molecules. (**B**) Effects of different concentrations of PEITC on mTOR, NF-κB, CCR7, PI3K, p-AKT, and AKT protein levels in CCRF-CEM cells, as determined by Western blotting. Quantitative data are presented as mean ± SD from three independent biological experiments (*n* = 3). Statistical significance was assessed using one-way ANOVA followed by Dunnett’s multiple-comparisons test. * *p* < 0.05, ** *p* < 0.01 and *** *p* < 0.001, compared to the control group.

The PI3K/AKT/mTOR signaling pathway plays a critical role in regulating leukemia cell growth, survival, metabolism, and proliferation [29]. Analysis of the PEITC-associated gene expression profile using IPA identified predicted changes involving PI3K, AKT, mTOR, NF-κB, and CCR7 within the cellular interaction network (Figure 3A). Based on these IPA predictions, Western blot analysis was performed to examine the protein levels of CCR7 and components of the PI3K/AKT/mTOR signaling pathway. Western blot analysis further showed that PEITC reduced PI3K and mTOR protein levels and markedly decreased AKT phosphorylation, whereas total AKT showed only a modest reduction. PEITC also reduced NF-κB and CCR7 protein levels in a concentration-dependent manner (Figure 3B). Together, these findings indicate that PEITC treatment modulates multiple components associated with the PI3K/AKT/mTOR signaling pathway in CCRF-CEM cells.

### 2.4. PEITC Caused Cell Cycle Perturbation and Microtubule Network Disruption

**Figure 4:**
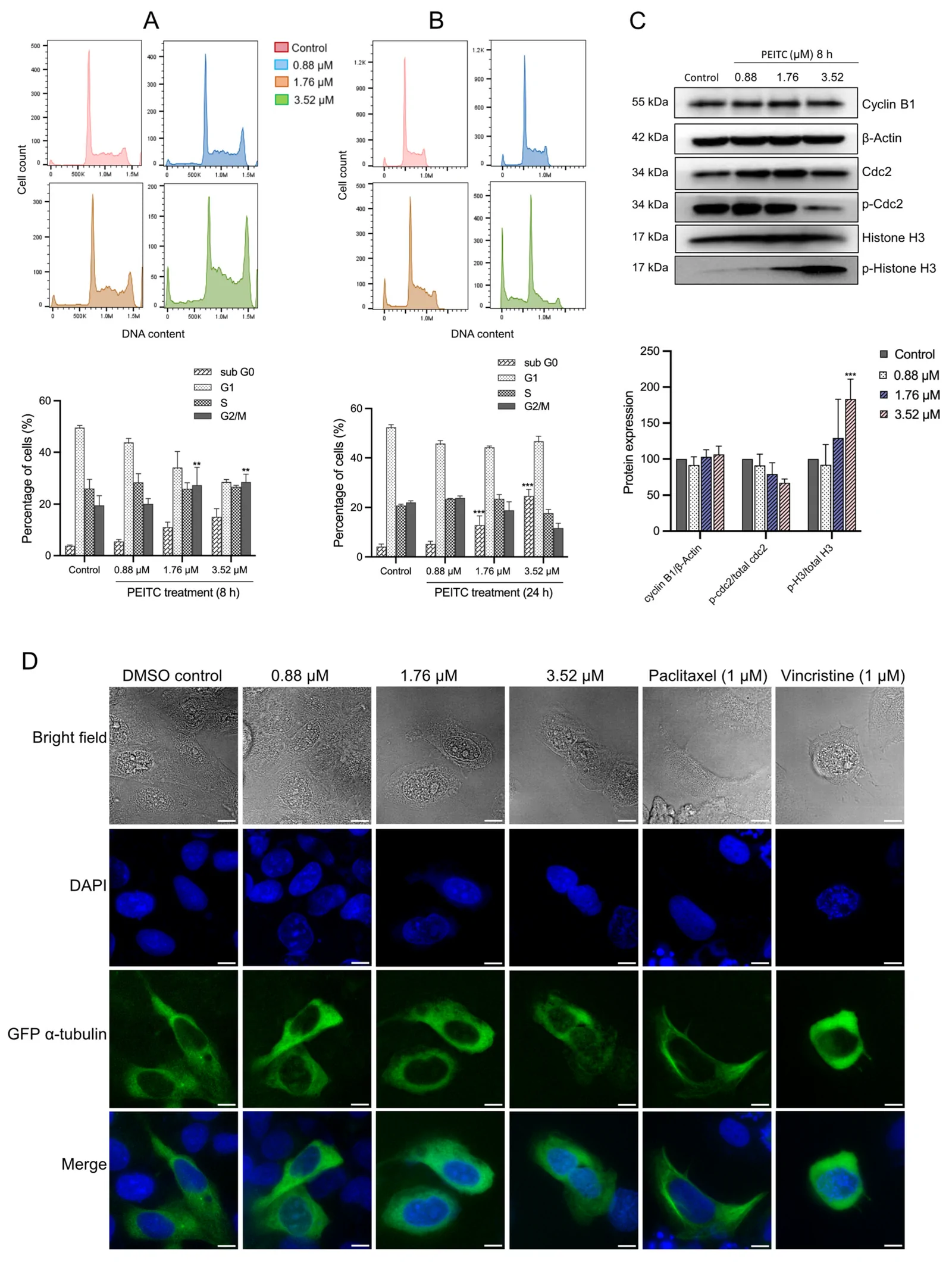
Effects of PEITC on cell cycle progression in CCRF-CEM cells and microtubule organization in U2OS cells stably expressing GFP-tagged-α-tubulin. Flow cytometric analysis of cell cycle distribution in CCRF-CEM cells treated with different concentrations of PEITC for 8 h (**A**) and 24 h (**B**). Quantitative data are presented as mean ± SD from three independent measurements (*n* = 3). Statistical significance was analyzed using one-way ANOVA followed by Dunnett’s multiple-comparisons test. ** *p* < 0.01, *** *p* < 0.001, compared to the control group. (**C**) Western blot analysis of cell cycle-related proteins, including cyclin B1, Cdc2, phosphorylated Cdc2 (p-Cdc2), histone H3 and phosphorylated histone H3 (p-H3) in CCRF-CEM cells, following 8 h of PEITC treatment. Protein levels were quantified by densitometric analysis, and data are presented as mean ± SD from three independent measurements (*n* = 3). Statistical significance was analyzed using one-way ANOVA followed by Dunnett’s multiple-comparisons test. *** *p* < 0.001, compared to the control group. (**D**) Representative fluorescence microscopy images of U2OS cells stably expressing GFP-tagged α-tubulin following treatment with PEITC or DMSO (control) for 24 h. GFP-tagged α-tubulin is shown in green, and nuclei were counterstained with 4′,6-diamidino-2-phenylindole (DAPI) (blue). Images were acquired using an AF7000 widefield fluorescence microscope at 65× magnification (scale bars = 50 µm).

Dysregulation of the cell cycle is a fundamental hallmark of cancer, making it a critical target for therapeutic intervention [30]. Alteration of the cell cycle emerged as one of the important cellular functions identified through IPA-based network analysis, prompting further biological validation. Therefore, to further elucidate the mechanism underlying PEITC-induced cytotoxicity, we investigated its effect on cell cycle distribution using flow cytometric analysis. Treatment of CCRF-CEM cells with PEITC for 8 h resulted in the accumulation of cells in the G2/M phase, indicating perturbation of cell cycle progression at G2/M. In contrast, prolonged exposure to PEITC for 24 h resulted in a significant increase in the sub-G0/G1 cell population (Figure 4A,B). To further characterize the G2/M perturbation observed at 8 h, Western blot analysis of cell cycle-related proteins was performed at this time point (Figure 4C). Exposure to PEITC resulted in a concentration-dependent increase in phosphorylated H3 at Ser10 and a decrease in the inhibitory phosphorylation of Cdc2/CDK1 at Tyr 15. In contrast, PEITC did not affect the expression of cyclin B1. These findings suggest increased representation of mitotic cells following PEITC treatment at 8 h.

Microtubules are essential structural components required for the proper chromosome segregation during mitosis [31]. Given the observed G2/M cell cycle perturbation and changes in mitosis-associated markers following PEITC treatment, we next performed a preliminary investigation of the effects of PEITC on microtubule organization. Fluorescence microscopy was performed using green fluorescent protein (GFP)-tagged α-tubulin-transfected human U2OS cells, which allows direct visualization of the microtubule network. Microtubule morphology was compared with that observed following treatment with the established antimitotic agents, vincristine and paclitaxel (Figure 4D). Control cells exhibited a well-defined microtubule network spreading throughout the cytoplasm from the nucleus. As expected, vincristine markedly disrupted the microtubule network, whereas paclitaxel produced dense and stabilized microtubule structures. PEITC treatment induced a dose-dependent alteration in microtubule organization. At higher concentrations, PEITC caused condensation and perinuclear localization of the microtubule network, with morphological changes resembling those observed in vincristine-treated cells.

### 2.5. PEITC Induced DNA Damage and Mitochondrial Dysfunction

**Figure 5:**
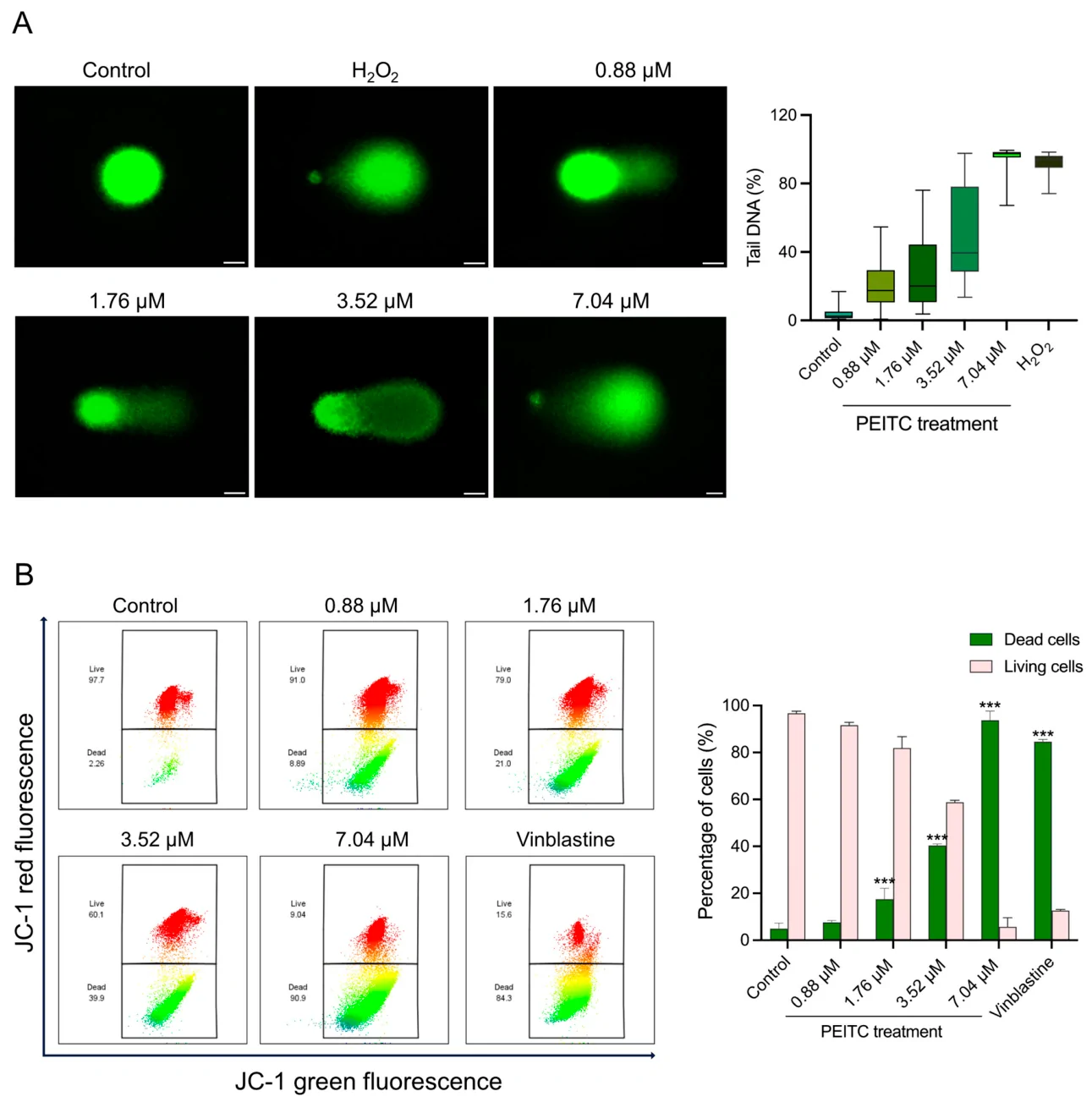
Analysis of DNA damage and mitochondrial membrane potential in PEITC-treated CCRF-CEM cells. (**A**) Representative images of the alkaline comet assay in CCRF-CEM cells treated with different concentrations of PEITC for 24 h. DNA damage was assessed as the percentage of DNA in the comet tail (% tail DNA) from 50 randomly selected comets per treatment group, as presented in the box plot. (**B**) Flow cytometric analysis of MMP in CCRF-CEM cells treated with different concentrations of PEITC or vinblastine for 24 h. The red and green colors represent live and dead cells, respectively. The percentage of dead cells exhibiting MMP loss was quantified. Data are presented as mean ± SD from three independent measurements (*n* = 3). Statistical significance was analyzed using one-way ANOVA followed by Dunnett’s multiple-comparisons test. *** *p* < 0.001, compared with the control group.

Microarray analysis revealed the enrichment of pathways associated with DNA damage repair in tumor cell lines. Previous studies have also reported that PEITC induces DNA damage and DNA strand breaks [17]. Therefore, the genotoxic effect of PEITC in CCRF-CEM cells was investigated using the alkaline comet assay. During electrophoresis, damaged DNA fragments migrate away from the nucleus, resulting in the characteristic “comet tails”. As shown in Figure 5A, PEITC treatment resulted in a clear, dose-dependent increase in DNA damage. Cells treated with 7.04 µM PEITC exhibited extensive DNA fragmentation, whereas untreated control cells showed minimal comet tail formation. The positive control, H_2_O_2,_ produced a marked increase in DNA damage. Quantitative analysis of 50 randomly selected comets per treatment group demonstrated a significant increase in % tail DNA following PEITC treatment.

DNA damage activates intracellular stress pathways that frequently converge on the mitochondria and cause a collapse in mitochondrial membrane potential [32]. We next investigated whether PEITC treatment affected mitochondrial membrane potential using JC-1 staining. In cells with polarized mitochondria, JC-1 accumulates within the mitochondria and forms aggregates that exhibit red fluorescence, whereas mitochondrial depolarization results in an increased proportion of JC-1 monomers exhibiting green fluorescence. As shown in Figure 5B, PEITC treatment caused a significant, dose-dependent loss of mitochondrial membrane potential in CCRF-CEM cells. Quantitative analysis demonstrated that 24 h of PEITC treatment significantly increased the proportion of cells exhibiting MMP loss, compared with the control. This effect was particularly pronounced at 7.04 µM, where 93.70 ± 3.93% of cells exhibited MMP loss. The positive control, vinblastine, consistently induced mitochondrial membrane depolarization.

### 2.6. PEITC Induced Apoptosis in CCRF-CEM Cells

**Figure 6:**
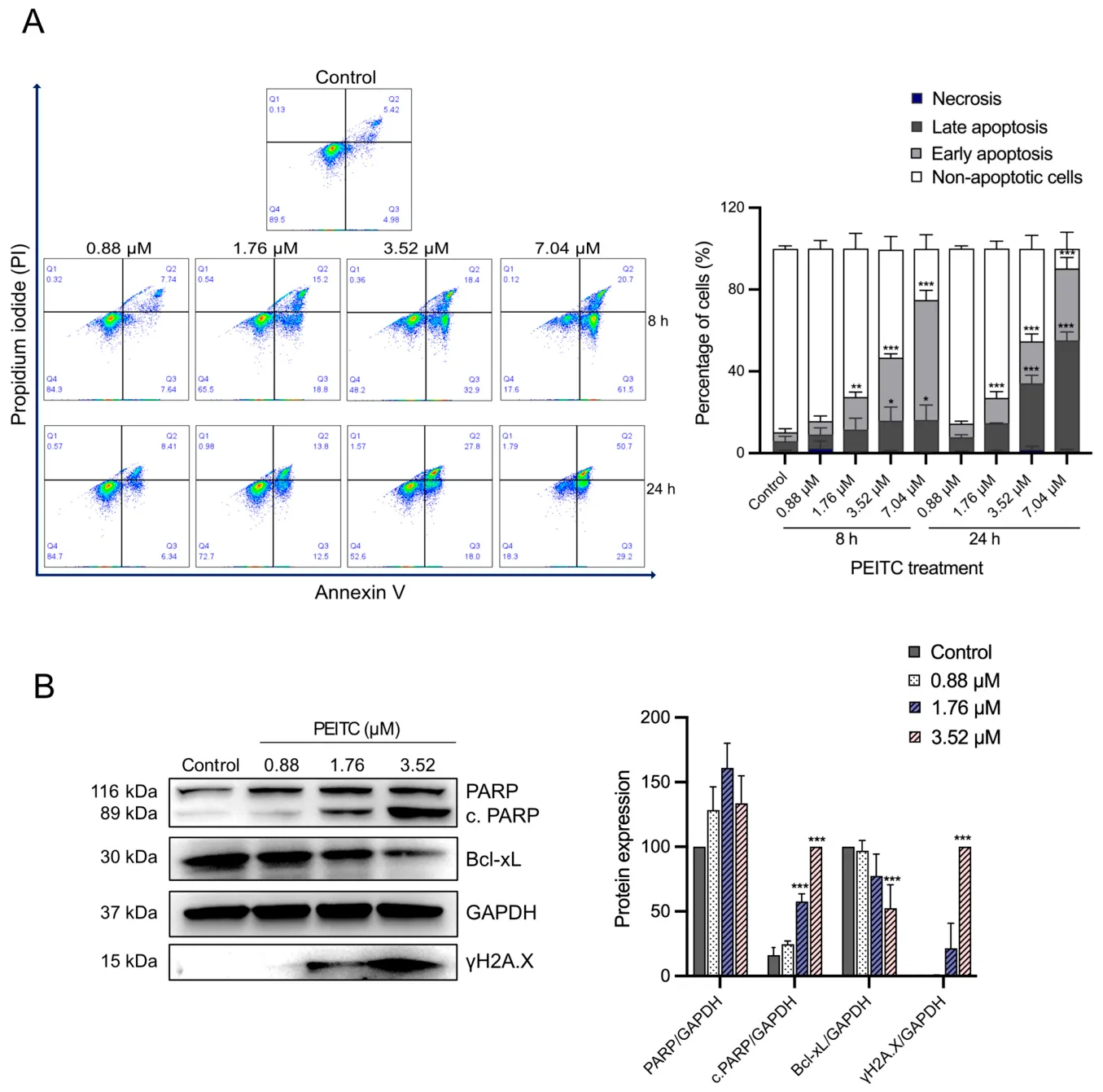
Assessment of apoptosis in PEITC-treated CCRF-CEM cells. (**A**) Apoptosis was assessed by flow cytometry using Annexin V-PI double staining. Q1 represents necrotic cells (Annexin V−/PI+), Q2 depicts late apoptotic cells (Annexin V+/PI+), Q3 displays early apoptotic cells (Annexin V+/PI−), and Q4 illustrates non-apoptotic cells (Annexin V−/PI−). The bar graphs show quantitative data presented as mean ± SD from three independent biological experiments (*n* = 3). (**B**) Effect of PEITC on PARP, Bcl-xL, and γH2A.X protein levels in CCRF-CEM cells as determined by Western blot. Quantitative data are presented as the mean ± SD of three independent biological experiments (*n* = 3). Statistical significance was assessed using one-way ANOVA followed by Dunnett’s multiple-comparisons test. * *p* < 0.05, ** *p* < 0.01, *** *p* < 0.001, compared to the control group.

The observed DNA damage and mitochondrial membrane depolarization following PEITC treatment suggest that apoptosis may contribute to the PEITC-induced cytotoxicity. Therefore, apoptosis was assessed by Annexin V-FITC/PI staining followed by flow cytometric analysis. PEITC treatment resulted in a concentration-dependent increase in apoptotic cell populations, as evidenced by increases in both early apoptotic (Annexin V^+^/PI^−^) and late apoptotic (Annexin V^+^/PI^+^) cells compared with the control group (Figure 6A). After 8 h of treatment, the early apoptotic cell population increased gradually, whereas after 24 h, the late-apoptotic cell population increased, indicating a time-dependent progression of the apoptotic response.

The effects of PEITC on PARP and Bcl-xL protein levels were further examined by Western blotting to characterize the apoptotic response (Figure 6B). PEITC treatment decreased the expression of the anti-apoptotic protein Bcl-xL and increased cleaved PARP levels compared with the control group, providing additional evidence of apoptosis induction. Moreover, PEITC also increased γH2A.X levels, particularly at higher concentrations, consistent with DNA damage observed following PEITC treatment.

### 2.7. PEITC Induced Autophagic Response in CCRF-CEM Cells

**Figure 7:**
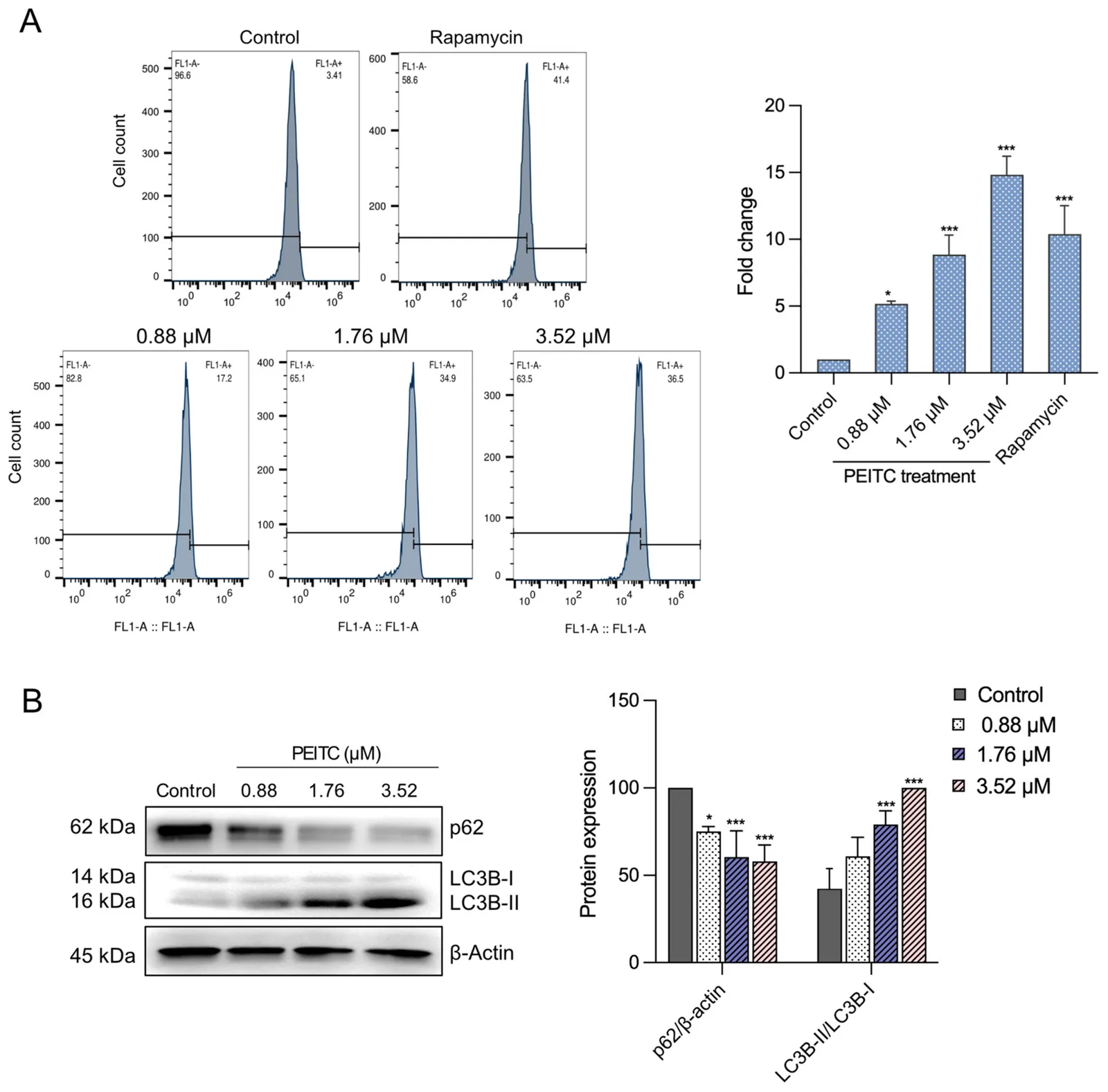
Autophagy-related changes in PEITC-treated CCRF-CEM cells. (**A**) Autophagy was assessed by flow cytometry in CCRF-CEM cells treated with different concentrations of PEITC for 24 h in the presence of chloroquine. Representative flow cytometry histograms and quantitative analysis are shown. The black line separates FL1-A− (autophagy-negative) and FL1-A+ (autophagy-positive) cell populations. Data are presented as mean ± SD of three independent biological experiments (*n* = 3). (**B**) Western blot analysis of p62 and LC3B, with quantitative analysis of p62 levels and the LC3B-II/LC3B-I ratio. Quantitative data are presented as the mean ± SD from three independent biological experiments (*n* = 3). Statistical significance was assessed using one-way ANOVA followed by Dunnett’s multiple-comparisons test. * *p* < 0.05, *** *p* < 0.001, compared to the control group.

Given the close association between mTOR signaling and the regulation of autophagy [33], we further investigated whether PEITC induces an autophagic response in CCRF-CEM cells. Autophagy-associated fluorescence was assessed by flow cytometry after 24 h of treatment (Figure 7A). The assay was performed in the presence of chloroquine, a lysosomal inhibitor. The positive control, rapamycin, showed a clear rightward shift in fluorescence intensity (FI). PEITC-treated cells also exhibited a concentration-dependent rightward shift in FI, indicating increased accumulation of the autophagy-associated signals.

Autophagy-related changes were further evaluated by Western blot analysis of LC3B and p62 (Figure 7B). PEITC treatment increased the LC3B-II/LC3B-I ratio and decreased p62 protein levels. Together, these findings support the induction of an autophagic response following PEITC treatment.

### 2.8. PEITC Inhibited Tube Formation in EA.hy926 Cells

**Figure 8:**
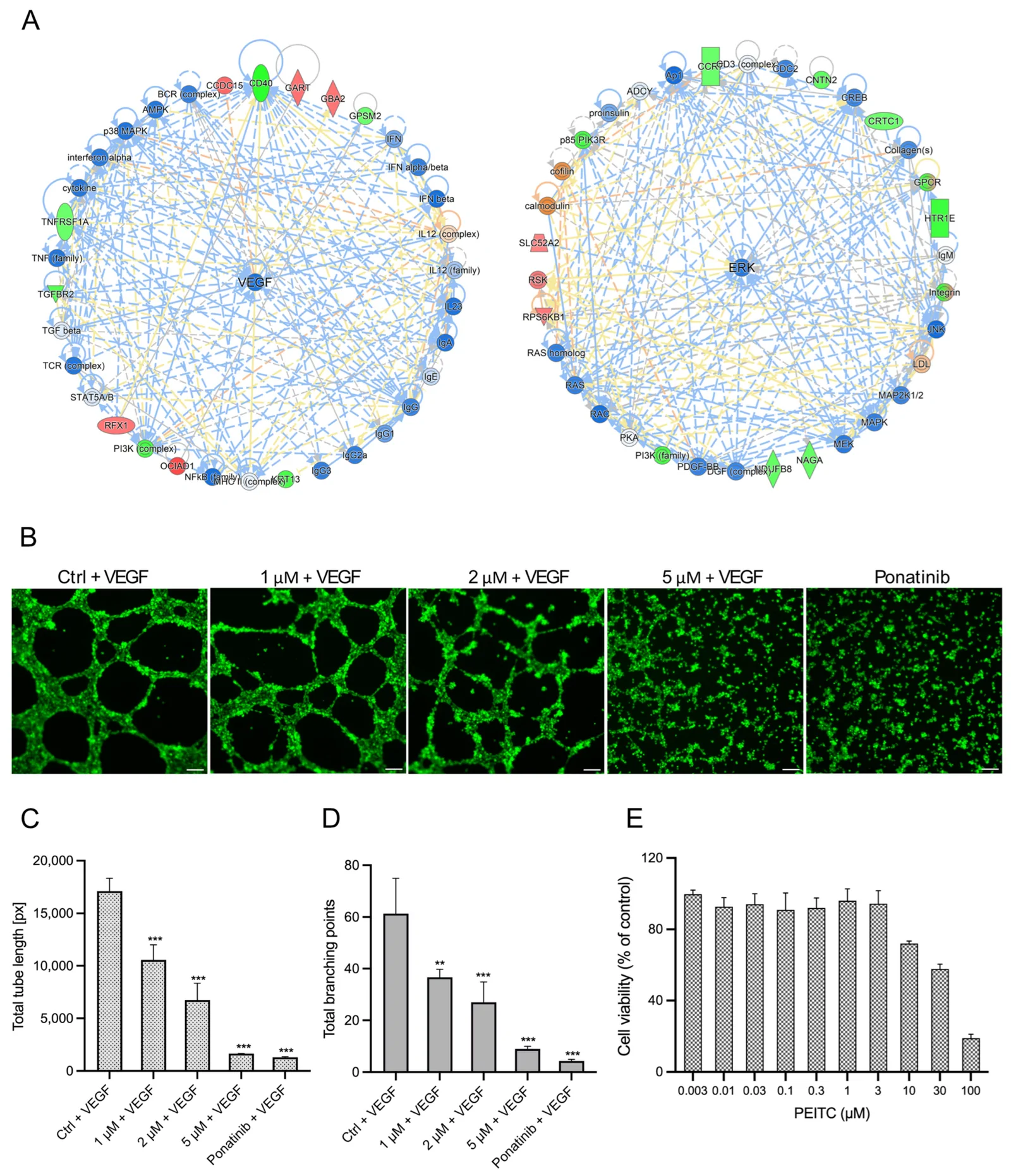
Effect of PEITC on VEGF-induced endothelial tube formation in EA.hy926 cells. (**A**) IPA-generated interaction network of regulated genes associated with VEGF signaling and angiogenesis-related processes. The blue and green indicate predicted inhibition or downregulation, whereas red and orange indicate predicted activation or upregulation, respectively. Solid lines indicate direct molecular relationships, whereas dashed lines represent indirect relationships between molecules. (**B**) Representative images of VEGF-induced endothelial tube formation following treatment with PEITC, DMSO control or ponatinib (positive control) for 12 h. (**C**,**D**) Quantitative analysis of total tube length and total branching points corresponding to the tube-formation assay shown in (**B**), analyzed using the Wimasis Image Analysis Platform. (**E**) Effect of PEITC on EA.hy926 cell viability. Data are presented as mean ± SD from three independent biological experiments (*n* = 3). Statistical significance was assessed using one-way ANOVA followed by Dunnett’s multiple-comparisons test. ** *p* < 0.01, *** *p* < 0.001, compared to control group.

The IPA analysis suggested that PEITC may modulate pathways associated with angiogenesis. (Figure 8A). Accordingly, the effect of PEITC on the vascular endothelial growth factor (VEGF)-induced endothelial tube formation was evaluated in EA.hy926 cells using a Matrigel-based tube formation assay, a widely used in vitro approach for assessing angiogenic activity [34]. PEITC disrupted VEGF-induced tube formation in a concentration-dependent manner. Quantitative analysis showed that PEITC significantly reduced both the total number of branching points and total tube length compared with the VEGF-treated control group. A similar inhibitory effect was observed with ponatinib, the positive control (Figure 8B–D). PEITC exhibited an IC_50_ of 38.20 ± 3.10 µM in EA.hy926 cells, substantially higher than the concentrations used in the tube-formation assay (1–5 µM) (Figure 8E). These findings demonstrate that PEITC inhibits VEGF-induced endothelial tube formation in EA.hy926 cells.

### 2.9. Antitumor Activity of PEITC in the Zebrafish Xenograft Model

**Figure 9:**
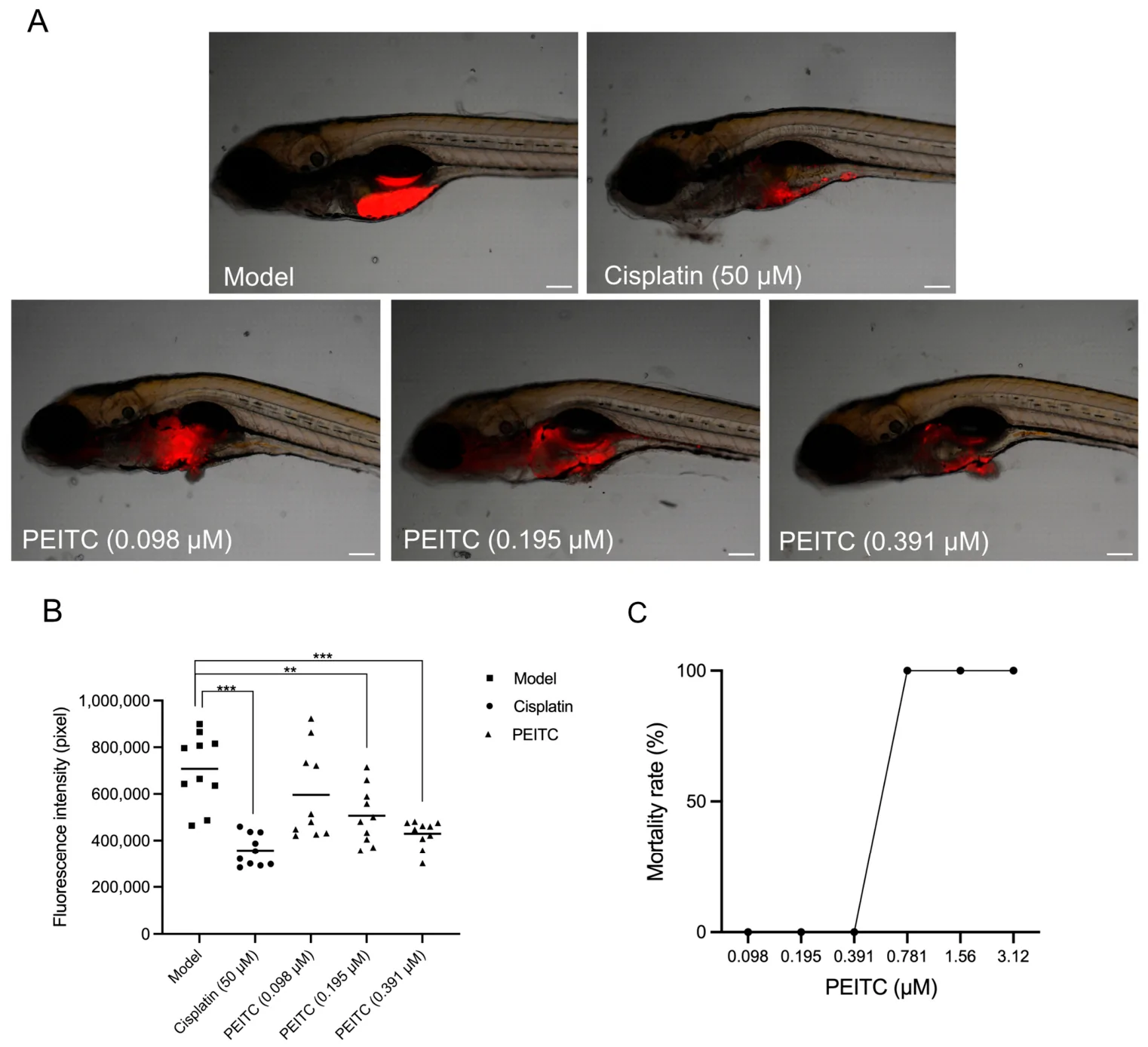
Assessment of PEITC-associated antitumor activity and toxicity in the CCRF-CEM zebrafish xenograft model. (**A**) Representative fluorescence images of CCRF-CEM xenografts in zebrafish following treatment with the indicated concentrations of PEITC or cisplatin as a positive control. Red fluorescence represents CM-DiI-labeled CCRF-CEM cells. Images were acquired at 60× magnification. (**B**) Quantification of tumor-associated fluorescence intensity. Data are presented as mean ± SD (*n* = 10 zebrafish per group). Statistical significance was assessed using one-way ANOVA followed by Dunnett’s multiple-comparisons test. ** *p* < 0.01, *** *p* < 0.001, compared to the model group. (**C**) PEITC-associated toxicity was assessed during the maximum tolerated concentration (MTC) experiment and expressed as mortality rate (%) (*n* = 30 zebrafish per group).

The preliminary antitumor activity of PEITC was evaluated using a zebrafish xenograft model established with CM-DiI-labelled CCRF-CEM cells. The zebrafish xenograft model is a rapid in vivo screening model for the preliminary assessment of antitumor activity. The absence of a fully developed adaptive immune system during the early larval stage of zebrafish facilitates the engraftment of malignant cells, while optical transparency enables direct visualization and monitoring of tumor growth and progression [35]. Cisplatin, used as a positive control, significantly reduced tumor-associated FI compared with the model control. PEITC treatment was associated with a concentration-dependent reduction in tumor-associated FI. Although a reduction was observed at 0.098 µM PEITC, the difference was not statistically significant compared with the model control. In contrast, treatment with 0.195 and 0.391 µM PEITC significantly reduced tumor-associated FI, consistent with reduced tumor burden at these concentrations. (Figure 9A,B). No mortality was observed in zebrafish at these PEITC concentrations, indicating that they were well tolerated under the experimental conditions (Figure 9C).

## 3. Discussion

The biological complexity of ALL is sustained by the interconnected signaling networks that regulate leukemia cell proliferation, survival, metabolism, and responses to cellular stress [36]. Despite advances in treatment and improved clinical outcomes, therapeutic resistance and disease relapse remain significant challenges, underscoring the need to explore alternative strategies that target multiple cancer-associated processes [37]. This is particularly relevant because the perturbation of one survival pathway can be compensated for by alternative signaling networks. Nature-derived bioactive compounds with pleiotropic activity may provide an opportunity to modulate multiple cellular processes simultaneously [38,39]. PEITC, an organosulfur compound, has been reported to affect cell proliferation, apoptosis, oxidative stress, signal transduction pathways, and epigenetic regulation in different cancer cell models [4]. However, its molecular and cellular effects in ALL have been insufficiently characterized, providing a rationale for the present investigation in CCRF-CEM T-ALL cells.

Previous studies have demonstrated the potential of PEITC to target resistant cancer phenotypes and overcome therapeutic resistance. For instance, PEITC has been reported to induce apoptosis in gefitinib-resistant NCI-H460 human lung cancer cells [40] and to reverse cisplatin resistance in NSCLC [20], suggesting that its activity may extend to certain drug-resistant cancer models. In the present study, PEITC exhibited potent cytotoxic activity against CCRF-CEM cells, with an IC_50_ of 1.76 µM. Similarly, Gao et al. reported PEITC-induced apoptosis in U937 leukemia cells at 4–8 µM after 3–6 h [41], supporting the activity of PEITC within the low-micromolar range across leukemia models, although differences in cell type, treatment duration, and experimental endpoint limit direct comparison. The activity of PEITC differed among the resistant sublines. CEM-ADR5000 cells showed reduced sensitivity to PEITC compared with parental CCRF-CEM cells, indicating cross-resistance. *P*-gp inhibition with verapamil did not significantly alter the intracellular accumulation of PEITC, suggesting that PEITC is unlikely to be a direct *P*-gp substrate [42]. The observed cross-resistance may therefore involve *P*-gp-independent mechanisms and require further investigation. Conversely, PEITC showed increased activity in BCRP-overexpressing cells and U87.MGΔEGFR cells relative to their corresponding parental cells. This enhanced sensitivity may reflect resistance-associated alterations in cellular processes that increase susceptibility to PEITC-induced cytotoxicity. Notably, normal PBMCs were approximately 4.4-fold less sensitive to PEITC than CCRF-CEM cells, indicating greater sensitivity of the leukemia cells under the condition tested. A similar pattern of selectivity has been reported previously, with primary CLL cells showing approximately five-fold greater sensitivity to PEITC than normal lymphocytes [43]. Although these findings suggest a degree of selectivity for malignant haematological cells, the magnitude observed in the present study was modest and should be interpreted with caution. Given the pronounced sensitivity of CCRF-CEM cells, we investigated the mechanism of action of PEITC in this model.

Among the various omics approaches currently available, microarray hybridization combined with IPA analysis provides a useful strategy for identifying molecular targets and affected signaling pathways [44]. In the present study, transcriptomic profiling using the combination of microarray and IPA revealed alteration of several canonical pathways following PEITC treatment, which indicates broad regulatory effects of PEITC on tumor-associated signaling networks. PI3K/AKT/mTOR signaling, a pathway critically involved in cancer cell survival, proliferation, and therapy resistance, was the top signaling pathway affected by PEITC treatment [26]. Consistent with our findings, previous studies have reported modulation of AKT-associated survival signaling by PEITC in hepatocellular carcinoma and leukemia models [41]. The genetic background of T-ALL may further influence the response to PEITC. PI3K/AKT/mTOR signaling and NOTCH1 are closely interconnected in T-ALL, while loss or inactivation of PTEN can promote sustained PI3K/AKT signaling and alter responses to targeted therapies [45,46]. Alterations in NOTCH1 or PTEN could therefore contribute to differences in PEITC sensitivity, particularly in the context of the signaling changes observed in the present study. However, this possibility was not directly investigated and should be examined using genetically defined or isogenic ALL models with different NOTCH1 and PTEN backgrounds. Moreover, in our study, we observed downregulation of CCR7 based on transcriptomic profiling. CCR7 contributes to leukemic cell survival, migration, and tissue homing and can activate downstream signaling pathways, including PI3K/AKT [47,48]. The reduced CCR7 expression following PEITC treatment may indicate attenuation of signaling processes that support T-ALL cell survival and trafficking. CCR7 downregulation could also potentially complement the cytotoxic effects of PEITC by reducing pro-survival signaling of leukemic cells. However, CCR7-dependent signaling warrants further assessment, and these implications remain to be experimentally established.

Tumorigenesis involves dysregulation of multiple interconnected signaling networks, supporting the rationale for therapeutic strategies that simultaneously target multiple cancer-associated processes [49]. In this context, the effects of PEITC on cell-cycle regulation, microtubule organization, DNA integrity, and cell death may reflect its broader cellular activity. PEITC induced an early G2/M cell-cycle perturbation in CCRF-CEM cells accompanied by an increase in the representation of mitotic cells. Entry into mitosis is mainly controlled by the Cyclin B1/CDK1 complex [50]. During G2, CDK1 activity is restrained by inhibitory phosphorylation at Tyr15 and Thr14, mediated by Wee1 and Myt1 kinases. At the G2/M transition, removal of these inhibitory phosphates by CDC25 phosphatases promotes CDK1 activation and mitotic entry [51]. In the present study, PEITC decreases the inhibitory phosphorylation of CDK1 at Tyr15 without altering cyclin B1 expression, suggesting that PEITC affects the regulatory CDK1 rather than cyclin B1 abundance. PEITC further increased histone H3 phosphorylation at Ser10, consistent with an increased representation of cells undergoing mitosis. Histone H3 phosphorylation is closely associated with chromosome condensation and widely used as a marker of mitosis [52]. However, additional mitotic markers, such as MPM-2 and PLK1, could further elucidate the effects of PEITC on mitotic entry and progression [53]. Notably, this early G2/M perturbation was followed by a pronounced increase in apoptosis at 24 h. These observations suggest that impaired mitotic progression may contribute to the subsequent apoptotic response. Further investigation of these regulatory markers at later time points would therefore be valuable for determining whether the mitotic phenotype observed at 8 h persists, resolves, or transitions to apoptosis.

The alterations in microtubule organization can interfere with mitotic spindle function and chromosome segregation. Given the observed G2/M cell cycle perturbation, we investigated whether PEITC also affects microtubule organization. Microtubules are highly dynamic cytoskeletal structures essential for proper mitotic spindle formation and chromosome segregation during cell division [54]. As a preliminary exploration of the effects of PEITC on microtubule organization, fluorescence imaging was performed in GFP-tagged α-tubulin-expressing U2OS cells, which provide a suitable model for visualization of the microtubule network. PEITC markedly altered microtubule organization in U2OS cells. However, the use of a different cell model represents a limitation, and these findings cannot be directly extrapolated to CCRF-CEM leukemia cells without further validation. Previous studies have identified tubulin as an intracellular target of ITCs, including PEITC, and demonstrated that these compounds can covalently modify tubulin and interfere with its polymerization [55,56]. Our findings are consistent with these previous observations, although direct binding of PEITC to tubulin was not examined in the present study. The potential involvement of microtubule-regulatory proteins, such as stathmin, also remains to be investigated. Further validation in CCRF-CEM or other leukemia models to determine whether similar microtubule alterations are associated with PEITC-induced mitotic accumulation will be an important direction for future research. Moreover, combining direct binding assays with analyses of microtubule-regulatory proteins will help further define how PEITC affects the microtubule network.

In light of the observed effects of PEITC on PI3K/AKT/mTOR signaling, cell-cycle progression, and microtubule organization, these findings also provide a rationale for investigating PEITC in combination with pathway inhibitors or microtubule-targeting agents. However, the present study evaluated PEITC as monotherapy, and therefore, no conclusions can be drawn regarding potential synergistic or additive effects of such combinations. Future studies could investigate whether combining PEITC with agents such as idelalisib or vincristine enhances anticancer activity or influences drug resistance in both drug-sensitive and drug-resistant leukemia models.

Previous studies have reported the DNA-damaging effects of ITCs, including PEITC in prostate and breast cancer cells [57,58]. Similarly, pronounced DNA damage was observed following PEITC treatment in CCRF-CEM cells, as evidenced by comet tail formation and elevated γH2A.X levels. These findings complement the microarray results showing enrichment of DNA damage repair-associated pathways and suggest that genotoxic stress is an important component of the cellular response to PEITC. Although γH2A.X is widely used as a marker of DNA damage response and is strongly associated with DNA double-strand breaks (DSBs) [59], its induction alone does not provide definitive evidence of DSB formation. Moreover, the alkaline comet assay detects DNA strand breaks and alkali-labile sites but does not specifically distinguish DSBs from other forms of DNA damage [60]. Therefore, the present findings support the induction of DNA damage by PEITC but do not establish the specific nature of the DNA lesions. Employing DSB-specific approaches, such as a neutral comet assay and analysis of 53BP1 nuclear foci, will be our main direction of future research to determine whether PEITC-induced DNA damage involves DSB formation. DNA damage can activate cell-cycle checkpoints that regulate G2/M progression, while persistent or unrepaired DNA damage can promote a shift toward apoptosis through activation of pro-apoptotic signaling [61]. In the present study, PEITC treatment was associated with concomitant MMP loss and an increase in apoptotic cell populations. These changes were accompanied by decreased expression of the anti-apoptotic protein Bcl-xL and increased PARP cleavage. Bcl-xL plays an important role in maintaining mitochondrial integrity and suppressing mitochondrial apoptotic signaling [62]. Therefore, its reduction is consistent with the alteration of mitochondrial apoptotic signaling in the cellular response to PEITC. Nevertheless, the temporal and causal relationship between mitochondrial depolarization and apoptosis cannot be established from the present findings. Mitochondrial dysfunction may occur upstream and contribute to apoptotic commitment, whereas loss of mitochondrial membrane potential can also develop downstream of caspase activation as part of the execution phase of apoptosis [63]. Alternatively, the observed alterations in DNA integrity, mitochondrial function, and apoptotic signaling may occur as interconnected components of a broader cellular stress response to PEITC. Further analysis of mitochondrial apoptotic markers and pathway inhibition is needed to determine whether mitochondrial dysfunction precedes or results from PEITC-induced apoptosis.

IPA revealed modulation of mTOR-related signaling following PEITC treatment, prompting further investigation of autophagy, as mTOR is a major regulator of this process [64]. PEITC increased autophagy-associated fluorescence and the LC3B-II/LC3B-I ratio while decreasing p62 levels, consistent with an autophagy-associated response. Similarly, previous studies have shown that glucosinolate derivatives can induce autophagy, often in association with mTOR inhibition and regulation of stress-related proteins such as Beclin1 and LC3B [65]. Autophagy may act as an adaptive mechanism by removing damaged proteins and organelles, thereby limiting further cellular stress. Conversely, under conditions of persistent or severe stress, sustained autophagic activity may accompany or contribute to cell death [66]. The functional role of this autophagic response to PEITC, however, remains unclear. Whether autophagy protects cells from PEITC-induced stress or contributes to cell death warrants further investigation. Targeting key autophagy-related genes, such as ATG5 or ATG7, may help distinguish between these two possibilities [67].

Angiogenesis supports tumor progression by supplying nutrients, oxygen, and survival signals to malignant cells, and its inhibition has important therapeutic effects [68]. Previous studies have shown that PEITC exhibits anti-angiogenic effects through HIF-1α and VEGF signaling [69,70]. In the present study, IPA predicted inhibition of angiogenesis-related processes, and the tube-formation assay showed that PEITC reduced endothelial cells’ ability to form capillary-like networks in vitro. However, the tube-formation assay is an in vitro surrogate for angiogenesis and does not fully recapitulate the complexity of blood vessel formation in vivo [71]. The present findings should be interpreted as evidence that PEITC interferes with endothelial network formation rather than as a direct demonstration of anti-angiogenic activity. Further validation using complementary angiogenesis models, including additional in vitro assays and in vivo approaches, would be required to establish PEITC’s anti-angiogenic potential.

Recently, the zebrafish tumor xenografts have emerged as a useful in vivo platform for investigating cancer cell proliferation, migration, and therapeutic responses [72]. Previous studies have demonstrated the anti-tumor activity of PEITC in several in vivo models, including rodent models [19,73]. Consistent with previous reports, PEITC significantly reduced tumor-associated FI in a zebrafish xenograft model. These findings provide preliminary in vivo evidence supporting the antileukemic activity of PEITC. However, the zebrafish xenograft model does not fully recapitulate the complexity of human leukemia or the systemic responses of mammalian organisms [74]. Validation in mammalian leukemia models is therefore important to establish the translational relevance of these findings.

The safety of PEITC in mammals needs to be considered, although several studies have reported minimal/mild adverse effects or cytotoxicity in animal models. For instance, in a 13-week study in F344 rats, dietary PEITC at 0.75–6 μmol/g diet was associated with minimal fatty changes in the liver, whereas a subsequent study reported dose-related hepatic morphological changes that varied with dietary conditions [75]. In a U937 leukemia xenograft model, PEITC at 50 mg/kg/day for 20 days inhibited tumor growth without significant body-weight loss or overt signs of toxicity [41]. Nevertheless, the available studies do not clearly define the dose-limiting toxicity of PEITC, particularly its effects on normal hematopoiesis. Further investigation in mammalian models, including hematological, biochemical, and histopathological assessments, will be important to characterize its safety profile and establish an appropriate therapeutic window.

Despite these promising findings, several limitations of the present study should be acknowledged. Firstly, the selectivity of PEITC toward leukemia cells relative to normal cells requires further investigation, particularly given the importance of preserving normal hematopoietic cells in the treatment of leukemia. The mechanistic investigations in the present study were performed in CCRF-CEM cells, which represent a T-ALL cell line. Whether PEITC exerts comparable effects in B-ALL remains to be established. Biological and molecular differences between T-ALL and B-ALL may contribute to differences in PEITC sensitivity. Additional work using B-ALL models, including NALM-6 and REH cells, is therefore warranted to evaluate the activity of PEITC across different ALL subtypes. Secondly, although the molecular analyses identified alterations in cancer-associated signaling pathways, further functional studies are required to establish a direct mechanistic relationship between these molecular changes and the observed cytotoxic effects. Thirdly, pharmacokinetic studies will also be important to determine whether therapeutically relevant PEITC exposure can be achieved and maintained in vivo. Fourthly, formulation may be an important consideration for the further development of PEITC. PEITC has previously been incorporated into liposomal and lipid nanoparticle systems for controlled release and targeted delivery [7,76]. Such approaches may offer opportunities to improve PEITC delivery in vivo and potentially enhance its therapeutic performance. In addition, prodrug strategies could be explored to improve its stability and pharmacokinetic properties. Collectively, further studies addressing selectivity, systemic safety, pharmacokinetics, and delivery will be important for determining the therapeutic potential of PEITC in acute lymphoblastic leukemia.

## 4. Materials and Methods

### 4.1. Reagents and Antibodies

PEITC and dimethyl sulfoxide (DMSO) were purchased from Sigma-Aldrich (Taufkirchen, Germany). Doxorubicin, vincristine and paclitaxel were generously provided by the University Medical Center of Johannes Gutenberg University Mainz (Mainz, Germany). Ponatinib was purchased from Biomol GmbH (Hamburg, Germany). PEITC was dissolved in DMSO to prepare a 20 mM stock solution, which was stored at −20 °C. Prior to the experiment, the PEITC stock solution was freshly diluted to the required concentration in culture medium. To ensure stability, the stock solution was used within a month after its preparation. The final DMSO concentration did not exceed 0.5% (*v*/*v*) in all the experiments.

Primary antibodies against AKT (4691), p-AKT (Ser473) (4060), PI3 Kinase p85 (4257), PARP (9532), mTOR (2983), NF-κB p65 (8242), γH2A.X (Ser139) (9718), PARP (9542), LC3B (2775), mTOR (2972), Histone H3 (D1H2) (4499), p-Histone H3 (S10) (9701), Cyclin B1 (4138), Cdc2 (77055), p-Cdc2 (Y15) (4539) and Bcl-xL (2764), GAPDH (5174) and β-actin (4970) were purchased from Cell Signaling Technology Europe B.V. (Leiden, The Netherlands), and primary antibody against CCR7 was purchased from Abcam Limited (Cambridge, UK). The primary antibody against p62/SQSTM1 (18420-1-AP) was purchased from Proteintech (Planegg-Martinsried, Germany).

### 4.2. Cell Lines and Cell Culture

A panel of cancer cell lines with different cellular and drug-resistance characteristics was used to evaluate the cytotoxic activity of PEITC across different cancer models. The leukemia cell lines, CCRF-CEM and its corresponding drug-resistant CEM/ADR5000 subline, were received from Dr. Axel Sauerbrey (Children’s Hospital, University of Jena, Jena, Germany) and Prof. Dietrich Niethammer (Department of Hematology and Oncology, Children’s University Hospital, Tübingen, Germany) [77,78]. These cells were cultured in RPMI-1640 medium (Life Technologies GmbH, Darmstadt, Germany) supplemented with 10% (*v*/*v*) fetal bovine serum (FBS) (Life Technologies GmbH) and 1% (*v*/*v*) penicillin/streptomycin (Life Technologies GmbH). The CEM/ADR5000 cells were exposed to doxorubicin (5000 ng/mL) every two weeks to maintain a resistant phenotype.

Human wild-type HCT116 colon cancer cells (p53^+/+^) and knockout clones (p53^−/−^) were obtained from Dr. B. Vogelstein and H. Hermeking (Howard Hughes Medical Institute, Baltimore, MD, USA) [79,80]. Breast cancer cells transfected with a control vector (MDA-MB-231-pcDNA3) and the transfectant subline MDA-MB-231-BCRP clone 23 were provided by Prof. Douglas Ross (University of Maryland Greenebaum Cancer Center, Department of Medicine, University of Maryland School of Medicine, and the Baltimore Veterans Affairs Medical Center, Baltimore, MD, USA) [81]. Human glioblastoma multiforme cells U87.MG and cells transfected with an expression vector carrying EGFR cDNA with a deletion of exons 2–7 (U87.MG∆EGFR) were received from Dr. Marc Halatsch (Department of Neurosurgery, University of Göttingen, Göttingen, Germany) and Dr. W. K. Cavenee (Ludwig Institute for Cancer Research, San Diego, CA, USA) [82,83]. These cells were cultured in DMEM medium (Life Technologies GmbH) supplemented with 10% (*v*/*v*) FBS and 1% (*v*/*v*) penicillin/streptomycin. HCT116 p53^−/−^ and U87.MG∆EGFR cell lines were maintained with 400 µg/mL geneticin (Sigma-Aldrich, Taufkirchen, Germany) and 800 ng/mL geneticin for the MDA-MB-231-BCRP clone 23 at two-week intervals. Similarly, human U2OS osteosarcoma cells expressing α-tubulin GFP were cultured in DMEM medium supplemented with 10% (*v*/*v*) FBS and 1% (*v*/*v*) penicillin/streptomycin. EA.hy926 endothelial cells were cultured in DMEM medium supplemented with 10% (*v*/*v*) FBS and 1% (*v*/*v*) penicillin/streptomycin. All cell lines used in this study were maintained and used at passage numbers below 30 throughout the experiment. The isolation and culture of human PBMCs were performed as previously described [84].

### 4.3. Resazurin Assay

The resazurin reduction assay was performed to determine the anti-proliferative effects of PEITC as previously described [85]. Suspension cells (1 × 10^4^ cells/well) and adherent cells (5 × 10^3^ cells/well) were seeded into 96-well culture plates and treated with PEITC (0.003–100 μM). A final concentration of 0.5% DMSO was used as the solvent control. After 72 h, cells were incubated with 20 μL of a 0.01% (*w*/*v*) resazurin (Promega GmbH, Mannheim, Germany) at 37 °C for 4 h. Fluorescence was measured using an Infinite M2000 Pro^TM^ plate reader (Tecan Deutschland GmbH, Crailsheim, Germany) at excitation and emission wavelengths of 544 and 590 nm, respectively.

The assay was performed in three independent experiments, each with six technical replicates. Dose–response curves were generated by plotting cell viability (% of control) against PEITC concentration, and half-maximal inhibitory concentration (IC_50_) values were determined. The resistance ratio was calculated by dividing the IC_50_ values of the resistant cell line by those of its corresponding sensitive cell line.

### 4.4. Microarray-Based mRNA Expression Profiling

To investigate PEITC-induced changes in gene expression, microarray-based transcriptome profiling was performed in CCRF-CEM cells. Cells were treated with PEITC (1.76 μM) or DMSO as a negative control. Microarray analysis was performed using two biological replicates (*n* = 2). Total RNA isolation was performed using an InviTrap^®^ Spin Universal RNA Mini Kit (Invitek Molecular GmbH, Berlin, Germany) according to the manufacturer’s instructions. RNA concentration and purity were assessed using a NanoDrop spectrophotometer (Nanodrop Technologies, LLC, Wilmington, DE, USA), while RNA quality and integrity were evaluated using a 5200 Fragment Analyzer (Agilent Technologies, Santa Clara, CA, USA). Microarray hybridization was performed on Affymetrix GeneChips^®^ with the human Clariom S^TM^ assay (Affymetrix Inc., Santa Clara, CA, USA) at the Genomics and Proteomics Core Facility of the German Cancer Research Centre (DKFZ, Heidelberg, Germany). Differentially expressed genes were evaluated using the Chipster analysis platform (version 3.16.3) (CSC, Espoo, Finland) (http://chipster.csc.fi/, accessed on 21 February 2025). Genes were initially filtered based on the coefficient of variation (0.1), and differentially expressed genes were then identified using the empirical Bayes moderated *t*-test. To account for multiple hypothesis testing, *p*-values were adjusted using the Benjamini–Hochberg method to control the FDR, with an FDR-adjusted *p*-value < 0.05 considered statistically significant. To investigate molecular pathways and biological functions associated with the PEITC-induced changes in gene expression, the identified differentially expressed genes were subsequently analyzed using IPA (Ingenuity Systems Inc., Redwood City, CA, USA).

### 4.5. qRT-PCR

To validate the microarray analysis, five genes, including three top upregulated (*SNCG*, *PLSCR1* and *ASPG*) and two downregulated genes (*VCF2* and *POLR2J2*), were selected for subsequent validation by real-time qRT-PCR. The primers were designed using the NCBI Primer-BLAST website (https://www.ncbi.nlm.nih.gov/tools/primer-blast/, accessed on 20 April 2025) and verified using the Oligo Analyze Tool (https://eurofinsgenomics.eu/en/ecom/tools/oligo-analysis/, accessed on 20 April 2025) (Eurofins Genomics Germany GmbH, Ebersberg, Germany). The primer sequences are listed in Table 2. Total RNA was extracted using an InviTrap^®^ Spin Universal RNA Mini Kit (Invitek Molecular GmbH, Berlin, Germany). Reverse transcription was performed using Luna Script™ RT SuperMix Kit (New England Biolabs GmbH, Frankfurt, Germany). qRT-PCR was performed on CFX384TM (Bio-Rad Laboratories GmbH, Munich, Germany) using EvaGreen master mix (5 × Hot Start Taq EvaGreen^®^ qPCR Mix, Axon-Labortechnik GmbH, Kaiserslautern, Germany) with 40 cycles. *GAPDH* served as the reference gene for normalization and relative gene expression was determined using the 2^−(ΔΔCT)^ method [86].

### 4.6. Western Blot

Cells were seeded into 6-well plates and treated with PEITC or DMSO for 24 h. Protein extraction was performed using M-PER^TM^ Mammalian Protein Extraction Reagent freshly supplemented with 1% Halt^TM^ Protease Inhibitor Cocktail and phosphatase inhibitor (all from Thermo Fisher Scientific Inc., Waltham, MA, USA). Following centrifugation, the proteins in the supernatant were stored at −80 °C. Equal amounts of protein were separated by SDS-PAGE gel and transferred to a polyvinylidene difluoride membrane. Blocking was performed with 5% BSA for 1 h, followed by incubation with primary antibodies overnight at 4 °C, then with secondary antibodies for 1 h. Protein bands were detected using Luminata™ Classico Western HRP substrate (Merck KGaA, Darmstadt, Germany) and visualized using an Alpha Innotech FluorChem Q system (Biozym Scientific GmbH, Oldendorf, Germany). Band intensities were quantified using ImageJ version 1.54i (National Institutes of Health, Bethesda, MD, USA) [87].

### 4.7. DNA Damage

DNA damage was evaluated using an alkaline comet assay with Oxiselect™ Comet Assay Kit (3-Well Slides) (BioCat GmbH, Heidelberg, Germany), adhering to the manufacturer’s protocol [88]. In brief, CCRF-CEM cells treated with PEITC or DMSO control for 24 h were collected, resuspended in phosphate-buffered saline (1 × 10^5^ cells/mL), and mixed with molten agarose (1:6) and applied to comet slides. Following solidification, the slides were lysed for 1 h and incubated in alkaline electrophoresis buffer for 40 min. Electrophoresis was performed at 20 V and 300 mA for 20 min. The slides were subsequently rinsed, dehydrated with 70% ethanol, air-dried, and stained with Vista Green DNA dye included in the kit. Comets were visualized using an EVOS digital inverted microscope (Life Technologies GmbH, Darmstadt, Germany). Fifty randomly selected comets per treatment group were analyzed using OpenComet in ImageJ version 1.54i, and DNA damage was quantified as the percentage of DNA in the comet tail (% tail DNA).

### 4.8. Mitochondrial Membrane Potential Assessment

Mitochondrial membrane potential (MMP) was assessed using the JC-1 mitochondrial membrane potential assay kit (Cayman Chemical Company, Ann Arbor, MI, USA) in accordance with the manufacturer’s instructions [89]. Cells were treated with PEITC for 24 h, with DMSO serving as a vehicle control and vinblastine as a positive control for mitochondrial depolarization. Following treatment, cells were incubated with JC-1 reagent at 37 °C for 15 min, washed, and resuspended in assay buffer. Flow cytometric analysis was performed using a Novocyte Quanteon flow cytometer (Agilent Technologies, Santa Clara, CA, USA). Debris was excluded by FSC-A/SSC-A gating, and singlets were selected using FSC-A/FSC-H. MMP was assessed in the singlet population using JC-1 aggregates and JC-1 monomers. In healthy cells, JC-1 forms J-aggregates that emit red fluorescence, which was detected using a 586/15 nm bandpass filter. JC-1 remains predominantly monomeric (in dead cells) and emits green fluorescence, which was detected using a 530/30 nm bandpass filter. Data were analyzed using FlowJo software version 10.10.0 (Becton, Dickinson and Company, Franklin Lakes, NJ, USA).

### 4.9. Cell Cycle Analysis

The cell cycle analysis was performed using propidium iodide (PI) staining as previously described [90]. CCRF-CEM cells were treated with different concentrations of PEITC or DMSO control for 8 and 24 h. Following treatment, cells were fixed with cold 80% ethanol and stored at −20 °C. Afterwards, cells were washed and incubated with 20 µg/mL RNase (Roche Deutschland Holding GmbH, Mannheim, Germany) for 1 h, followed by PI staining (50 µg/mL). PI staining was measured using a BD Accuri^TM^ C6 Flow Cytometer (Becton, Dickinson and Company, Franklin Lakes, NJ, USA), and the data were analyzed using FlowJo software version 10.10.0. Cells were initially gated on FSC-A/SSC-A to exclude debris, and doublets were excluded using FSC-A/FSC-H, thereby restricting the analysis to singlet cells. DNA content of the resulting singlet population was determined from PI fluorescence detected on the FL2-A channel.

### 4.10. Fluorescence Microscopy of α-Tubulin GFP

Human U2OS osteosarcoma cells expressing α-tubulin GFP (3 × 10^4^ cells/well) were cultured overnight in µ-Slide 8 Well (Ibidi GmbH, Gräfelfing, Germany), followed by 24 h exposure to PEITC, DMSO control, 1 µM vincristine, or 1 µM paclitaxel. Cells were fixed with 4% paraformaldehyde and stained with 1 µg/mL DAPI (Sigma-Aldrich, Merck KGaA, Darmstadt, Germany). Fluorescence images were acquired using an AF7000 widefield fluorescence microscope (Leica Microsystems GmbH, Wetzlar, Germany) and analyzed with Fiji ImageJ version 1.54p (National Institutes of Health, Bethesda, MD, USA).

### 4.11. Apoptosis Assay

Apoptosis was analyzed using the Annexin V/PI double-staining kit (Invitrogen eBioscience^TM^; Thermo Fisher Scientific Inc., Waltham, MA, USA) according to the manufacturer’s protocol. CCRF-CEM cells were collected after 8 and 24 h of treatment with PEITC or DMSO, stained with Annexin V for 15 min, and subsequently with PI. Samples were analyzed using a BD Accuri^TM^ C6 Flow Cytometer, and the data were analyzed using FlowJo software version 10.10.0. Debris and doublets were excluded by FSC-A/SSC-A and FSC-A/FSC-H. Annexin V and PI fluorescence were detected in the FL1 (530/30 nm) and FL2 (610/20 nm) channels, respectively.

### 4.12. Autophagy Detection

Autophagy was assessed using the Autophagy Detection Kit (Abcam Limited, Cambridge, UK) according to the manufacturer’s instructions. CCRF-CEM cells were treated with PEITC, DMSO control or rapamycin (500 nM) in the presence of chloroquine (50 µM) for 24 h. Cells were washed and incubated with green stain solution at 37 °C for 30 min and analyzed using a BD Accuri^TM^ C6 Flow Cytometer. Debris was excluded by FSC-A/SSC-A gating, followed by FSC-A/FSC-H gating to select singlets. Green fluorescence was detected on the FL1 (530/30 nm) channel. Data were analyzed using FlowJo software version 10.10.0.

### 4.13. Tube Formation Assay

The tube formation assay was performed using EA.hy926 endothelial cells to assess angiogenic activity, as previously described [91]. In brief, μ-slides were coated with 10 μL Matrigel^®^ and incubated for 30 min at 37 °C. Cells (2 × 10^4^ cells/mL) were seeded onto Matrigel^®^ and treated with PEITC, DMSO or ponatinib as a positive control for 12 h. Finally, the cells were stained with 6.25 μg/mL Calcein-AM (Cayman Chemical, Ann Arbor, MI, USA), and fluorescence images were captured with an AF7000 widefield fluorescence microscope (Leica Microsystems GmbH, Wetzlar, Germany). Total tube length and branching points were quantified using the Wimasis Image Analysis Platform (https://www.wimasis.com, accessed on 23 March 2026).

### 4.14. Zebrafish Xenograft

A wild-type AB strain of zebrafish was obtained from the China Zebrafish Resource Center, Institute of Hydrobiology, China Academy of Sciences (Wuhan, China). All zebrafish experiments were reviewed and approved by the Institutional Animal Care and Use Committee (IACUC) at Hunter Biotechnology Inc. Larvae obtained by natural spawning were maintained under standard conditions (14:10 h light/dark cycle) and fed fry flakes and brine shrimp. For MTC determination, 30 larvae per group were exposed to PEITC (0.195, 0.391, 0.781, 1.56, and 3.12 μM), and the adverse effects and mortality were monitored. For the tumor xenograft assay, CCRF-CEM cells were stained with CM-Dil (1:1000) and 200 cells were microinjected into the yolk sac of each larva at 24 h post-fertilization. Tumor establishment was verified using an AZ100 fluorescent microscope (Nikon Corporation, Tokyo, Japan). Xenografted larvae were subsequently exposed to PEITC or cisplatin for 48 h. Tumor FI was analyzed using NIS-Elements (Nikon Corporation, Tokyo, Japan), and tumor inhibition was calculated as [1 − (FI treated/FI control)] × 100%.

### 4.15. Statistical Analysis

Statistical analysis was performed using GraphPad 10.2.3 software (GraphPad, La Jolla, CA, USA). All measurements and results were presented as mean ± SD. Comparison between means was tested by Student’s *t*-test or one-way ANOVA, and *p* < 0.05 was considered statistically significant.

## 5. Conclusions

In summary, this study demonstrates that PEITC exerts multi-targeted anti-leukemic activity against acute lymphoblastic leukemia (ALL) cells in vitro and in vivo. PEITC inhibited the proliferation of various cancer cell lines, with particularly pronounced effects on CCRF-CEM cells. Microarray and IPA analyses provide insight into the mode of action of PEITC in CCRF-CEM cells. Specifically, PEITC modulated the PI3K/AKT/mTOR signaling axis, induced DNA damage, apoptosis, and autophagy-related changes. On the other hand, PEITC altered microtubule organization in GFP α-tubulin-expressing U2OS cells and inhibited VEGF-induced endothelial tube formation in EA.hy926 cells. PEITC also demonstrated preliminary antitumor activity in vivo in the zebrafish xenograft model. Although the mechanistic causality, selectivity, model-specific findings, and translational relevance require further validation, our results identify PEITC as a candidate for further preclinical investigation in ALL.

## Figures and Tables

**Table 1:** IC_50_ values of PEITC in cancer cell lines and normal leukocytes.

Cell Line	IC_50_, Mean ± SD (µM)	95% CI (µM)	Resistance Ratio
CCRF-CEM	1.76 ± 0.07	1.66–1.86	13.05
CEM/ADR5000	22.98 ± 6.82	10.82–32.21	
MDA-MB-231-pcDNA3	7.52 ± 0.27	6.84–8.19	0.71
MDA-MB-231-BCRP clone 23	5.39 ± 0.51	4.13–6.66	
HCT116 (p53^+/+^)	5.63 ± 3.04	−1.92–13.19	1.0
HCT116 (p53^−/−^)	5.66 ± 2.88	−1.51–12.83	
U87.MG	22.39 ± 3.44	13.83–30.94	0.49
U87.MG∆EGFR	11.06 ± 0.48	9.89–12.26	
PBMCs	7.61 ± 0.26	6.95–8.27	NA

NA: Not applicable.

**Table 2:** Primer’s sequences (5′→3′) for qRT-PCR.

Genes	Forward Primer	Reverse Primer
*SNCG*	GAAGGGCTTCTCCATCGCCA	TTCTCGGCCACTGAGGTCAC
*PLSCR1*	GGCAGCCAGAGACTCACAGA	GCTGGGCCAGGACCTGAATG
*ASPG*	GCACACCCCCGTCCACT	CGTGCTCCTCGTCATGGAAC
*VCF2*	CCCCACGGTCATCACGCTTT	TCCTTTTGGGCTGGGTGGAAT
*POLR2J2*	AGTCGTTCTTGCTCTTCGAGGG	AAGCAGGCCTTGGGTACCTT
*GAPDH*	GTTCGACAGTCAGCCGCATC	GGCGCCCAATACGACCAAAT

## Data Availability

The original contributions presented in this study are included in the article/Supplementary Material. Further inquiries can be directed to the corresponding author.

## Supplementary Materials

The following supporting information can be downloaded at: https://www.mdpi.com/article/10.3390/ph19101631/s1. Table S1: Downregulated and upregulated genes in CCRF-CEM leukemia cells following treatment with PEITC at the IC_50_ concentration for 24 h.

## Abbreviations

The following abbreviations are used in this manuscript:

ANOVA	Analysis of variance
ALL	Acute lymphoblastic leukemia
BCRP	Breast cancer resistance protein
DAPI	4′,6-diamidino-2-phenylindole
DMSO	Dimethyl sulfoxide
FBS	Fetal bovine serum
FDR	False discovery rate
FI	Fluorescence intensity
GFP	Green fluorescent protein
IPA	Ingenuity pathway analysis
ITCs	Isothiocyanates
MMP	Mitochondrial membrane potential
MTC	Maximum tolerated concentration
NSCLC	Non-small cell lung cancer
PBMCs	Peripheral blood mononuclear cells
PEITC	Phenethyl isothiocyanate
P-gp	P-glycoprotein
PI	Propidium iodide
qRT-PCR	Quantitative reverse transcription polymerase chain reaction
VEGF	Vascular endothelial growth factor
