# Supplementary material for: Mechanistic Profiling of Phenethyl Isothiocyanate in CCRF-CEM Acute Lymphoblastic Leukemia Cells
Source: Pharmaceuticals (Basel). 2026 Oct 14;19(10):1631. doi: 10.3390/ph19101631 (PMC13708131; doi:10.3390/ph19101631)
Supplement: Supplementary file 1 [file pharmaceuticals-19-01631-s001.zip › pharmaceuticals-4524659-supplementary.pdf]

Supplementary file

Supplementary Table S1: Downregulated and upregulated genes in CCRF-CEM leukemia cells following treatment with PEITC at the IC_{50} concentration for 24 h.

Symbol	Entrez Gene Name	Expr Fold Change
SLC16A1	solute carrier family 16 member 1	-3.079
VCF2	VCP nuclear cofactor family member 2	-1.73
SPIN4	spindlin family member 4	-1.727
POLR2J2/POLR2J3	RNA polymerase II subunit J2	-1.679
PPFIBP1	PPFIB scaffold protein 1	-1.677
USB1	U6 snRNA biogenesis phosphodiesterase 1	-1.639
CD40	CD40 molecule	-1.63
RIPK3	receptor interacting serine/threonine kinase 3	-1.618
MYH10	myosin heavy chain 10	-1.595
DHRS1	dehydrogenase/reductase 1	-1.591
ITGB5	integrin subunit beta 5	-1.539
SERTAD3	SERTA domain containing 3	-1.527
LYST	lysosomal trafficking regulator	-1.495
HTR1E	5-hydroxytryptamine receptor 1E	-1.494
HECA	hdc homolog, cell cycle regulator	-1.493
GTPBP1	GTP binding protein 1	-1.482
ZFTRAF1	zinc finger TRAF-type containing 1	-1.478
PIK3R2	phosphoinositide-3-kinase regulatory subunit 2	-1.455
KIF14	kinesin family member 14	-1.441
KRT13	keratin 13	-1.421
MCTP1	multiple C2 and transmembrane domain containing 1	-1.411
SERTAD2	SERTA domain containing 2	-1.405
CAST	calpastatin	-1.385
JMJD6	jumonji domain containing 6, arginine demethylase and lysine hydroxylase	-1.382
AK9	adenylate kinase 9	-1.373
MSH3	mutS homolog 3	-1.358
KLK14	kallikrein related peptidase 14	-1.35
DMXL1	Dmx like 1	-1.348
GOLGA8K (includes others)	golgin A8 family member K	-1.348
WDR6	WD repeat domain 6	-1.335
UBXN6	UBX domain protein 6	-1.334
TGFBR2	transforming growth factor beta receptor 2	-1.333
MICOS10-NBL1	MICOS10-NBL1 readthrough	-1.327
LRRC14B	leucine rich repeat containing 14B	-1.326
DUSP16	dual specificity phosphatase 16	-1.321
LINC00885	--	-1.319
ZNF91	zinc finger protein 91	-1.312
ZNF614	zinc finger protein 614	-1.308
PHLDB1	pleckstrin homology like domain family B member 1	-1.293
TMEM184B	transmembrane protein 184B	-1.267
RPN2	ribophorin II	-1.266
YIPF4	Yip1 domain family member 4	-1.264

BTD	biotinidase	-1.264
NKX6-2	NK6 homeobox 2	-1.264
SUSD3	sushi domain containing 3	-1.263
ZNHIT2	zinc finger HIT-type containing 2	-1.261
CNTN2	contactin 2	-1.255
KLF12	KLF transcription factor 12	-1.253
RALGDS	ral guanine nucleotide dissociation stimulator	-1.25
CYTH2	cytohesin 2	-1.247
SATB2	SATB homeobox 2	-1.246
PPP6R1	protein phosphatase 6 regulatory subunit 1	-1.242
HS3ST3B1	heparan sulfate-glucosamine 3-sulfotransferase 3B1	-1.233
RHPN1	rhophilin Rho GTPase binding protein 1	-1.23
DDX41	DEAD-box helicase 41	-1.23
NDUFB8	NADH:ubiquinone oxidoreductase subunit B8	-1.222
B4GALNT1	beta-1,4-N-acetyl-galactosaminyltransferase 1	-1.214
TMOD2	tropomodulin 2	-1.209
CEP89	centrosomal protein 89	-1.208
HYOU1	hypoxia up-regulated 1	-1.208
NIT2	nitrilase family member 2	-1.206
GMPPA	GDP-mannose pyrophosphorylase A	-1.202
AP2A1	adaptor related protein complex 2 subunit alpha 1	-1.202
DHRS11	dehydrogenase/reductase 11	-1.201
NAGA	alpha-N-acetylgalactosaminidase	-1.199
GBF1	golgi brefeldin A resistant guanine nucleotide exchange factor 1	-1.195
SZRD1	SUZ RNA binding domain containing 1	-1.191
SARS1	seryl-tRNA synthetase 1	-1.183
IFI27L2	interferon alpha inducible protein 27 like 2	-1.176
SETD1B	SET domain containing 1B, histone lysine methyltransferase	-1.175
FBXW7	F-box and WD repeat domain containing 7	-1.171
SLC12A4	solute carrier family 12 member 4	-1.17
LNX2	ligand of numb-protein X 2	-1.169
CRTAP	cartilage associated protein	-1.167
SLC9A1	solute carrier family 9 member A1	-1.166
MED18	mediator complex subunit 18	-1.166
SLC22A20P	solute carrier family 22 member 20, pseudogene	-1.162
WDR62	WD repeat domain 62	-1.162
RUSF1	RUS family member 1	-1.159
ZNF609	zinc finger protein 609	-1.156
SMURF1	SMAD specific E3 ubiquitin protein ligase 1	-1.156
IMPDH1	inosine monophosphate dehydrogenase 1	-1.154
CCR7	C-C motif chemokine receptor 7	-1.153
CAMK1	calcium/calmodulin dependent protein kinase I	-1.15
GPSM2	G protein signaling modulator 2	-1.149
TINF2	TERF1 interacting nuclear factor 2	-1.148
SATB1	SATB homeobox 1	-1.147
TNFRSF1A	TNF receptor superfamily member 1A	-1.146
ZDHHC5	zDHHC palmitoyltransferase 5	-1.144
CAPN1	calpain 1	-1.142
PSMD2	proteasome 26S subunit ubiquitin receptor, non-ATPase 2	-1.138
APMAP	adipocyte plasma membrane associated protein	-1.137
CDC123	cell division cycle 123	-1.137
CRTC1	CREB regulated transcription coactivator 1	-1.133

ECD	ecdysoneless cell cycle regulator	-1.132
PCNX1	pecanex 1	-1.128
ORAI2	ORAI calcium release-activated calcium modulator 2	-1.126
SIL1	SIL1 nucleotide exchange factor	-1.126
COL23A1	collagen type XXIII alpha 1 chain	-1.125
DUSP7	dual specificity phosphatase 7	-1.123
KCNH2	potassium voltage-gated channel subfamily H member 2	-1.113
NADSYN1	NAD synthetase 1	-1.112
MRRF	mitochondrial ribosome recycling factor	-1.112
AGAP5	ArfGAP with GTPase domain, ankyrin repeat and PH domain 5	-1.111
ARAF	A-Raf proto-oncogene, serine/threonine kinase	-1.106
ERVH48-1	endogenous retrovirus group 48 member 1, envelope	-1.106
SRSF4	serine and arginine rich splicing factor 4	-1.104
PHF2	PHD finger protein 2	-1.101
DNAJB6	DnaJ heat shock protein family (Hsp40) member B6	-1.099
PBX2	PBX homeobox 2	-1.098
TTC19	tetratricopeptide repeat domain 19	-1.098
AFTPH	aftiphilin	-1.097
ERAP2	endoplasmic reticulum aminopeptidase 2	-1.095
MPP1	MAGUK p55 scaffold protein 1	-1.091
STK25	serine/threonine kinase 25	-1.085
RPL8	ribosomal protein L8	-1.084
FBXL14	F-box and leucine rich repeat protein 14	-1.075
MRPL42	mitochondrial ribosomal protein L42	-1.068
ACTG1	actin gamma 1	-1.064
HNRNPUL1	heterogeneous nuclear ribonucleoprotein U like 1	-1.057
EIF4A1	eukaryotic translation initiation factor 4A1	-1.052
PSMD4	proteasome 26S subunit ubiquitin receptor, non-ATPase 4	-1.049
SRPRA	SRP receptor subunit alpha	-1.039
C4orf46	chromosome 4 open reading frame 46	1.041
SGTA	small glutamine rich tetratricopeptide repeat co-chaperone alpha	1.046
DHDDS	dehydrodolichyl diphosphate synthase subunit	1.072
CHURC1-FNTB	CHURC1-FNTB readthrough	1.074
ECHDC1	ethylmalonyl-CoA decarboxylase 1	1.078
URI1	URI1 prefoldin like chaperone	1.078
CBX3	chromobox 3	1.079
SLC52A2	solute carrier family 52 member 2	1.092
ADSS2	adenylosuccinate synthase 2	1.098
SRSF10	serine and arginine rich splicing factor 10	1.103
TMEM248	transmembrane protein 248	1.105
ARMC5	armadillo repeat containing 5	1.11
NCK1	NCK adaptor protein 1	1.112
WDCP	WD repeat and coiled coil containing	1.112
LRWD1	leucine rich repeats and WD repeat domain containing 1	1.113
RNF4	ring finger protein 4	1.115
MAP7D3	MAP7 domain containing 3	1.12
MARCHF7	membrane associated ring-CH-type finger 7	1.125
B4GALT5	beta-1,4-galactosyltransferase 5	1.136
POLR1E	RNA polymerase I subunit E	1.14
ITGAX	integrin subunit alpha X	1.142
CNBP	CCHC-type zinc finger nucleic acid binding protein	1.145
WWP2	WW domain containing E3 ubiquitin protein ligase 2	1.147

FAM135A	family with sequence similarity 135 member A	1.153
CUTA	cutA divalent cation tolerance homolog	1.158
HTATSF1	HIV-1 Tat specific factor 1	1.159
ORC5	origin recognition complex subunit 5	1.162
VPS26A	VPS26 retromer complex component A	1.163
H1-0	H1.0 linker histone	1.17
ARRDC1-AS1	ARRDC1 antisense RNA 1	1.171
TCTN2	tectonic family member 2	1.175
SCGB1C2	secretoglobin family 1C member 2	1.184
ZNF761	zinc finger protein 761	1.189
NFIC	nuclear factor I C	1.19
CDH22	cadherin 22	1.191
RPS6KB1	ribosomal protein S6 kinase B1	1.192
PBK	PDZ binding kinase	1.193
RARA	retinoic acid receptor alpha	1.193
CDH3	cadherin 3	1.193
ZNF805	zinc finger protein 805	1.194
MROH1	maestro heat like repeat family member 1	1.194
RFX1	regulatory factor X1	1.197
ROGDI	rogdi atypical leucine zipper	1.205
NUP35	nucleoporin 35	1.206
UTP14A	UTP14A small subunit processome component	1.21
GINS1	GINS complex subunit 1	1.21
GBA2	glucosylceramidase beta 2	1.212
TSR1	TSR1 ribosome maturation factor	1.216
USP1	ubiquitin specific peptidase 1	1.219
RECQL	RecQ like helicase	1.22
SYPL1	synaptophysin like 1	1.223
STX6	syntaxin 6	1.226
GART	phosphoribosylglycinamide formyltransferase, phosphoribosylglycinamide synthetase, phosphoribosylaminoimidazole synthetase	1.228
USP15	ubiquitin specific peptidase 15	1.238
COMTD1	catechol-O-methyltransferase domain containing 1	1.242
LANCL2	LanC like glutathione S-transferase 2	1.242
TTC5	tetratricopeptide repeat domain 5	1.246
CLDN18	claudin 18	1.249
KCNA3	potassium voltage-gated channel subfamily A member 3	1.262
MTFP1	mitochondrial fission process 1	1.262
ASAP1	ArfGAP with SH3 domain, ankyrin repeat and PH domain 1	1.268
NAMPT	nicotinamide phosphoribosyltransferase	1.268
KNL1	kinetochore scaffold 1	1.268
TTL	tubulin tyrosine ligase	1.269
MTURN	maturin, neural progenitor differentiation regulator homolog	1.277
JSRP1	junctional sarcoplasmic reticulum protein 1	1.28
FRA10AC1	FRA10A associated CGG repeat 1	1.286
ORC2	origin recognition complex subunit 2	1.291
CFAP97	cilia and flagella associated protein 97	1.292
LRRFIP1	LRR binding FLII interacting protein 1	1.302
ACADSB	acyl-CoA dehydrogenase short/branched chain	1.305
AK2	adenylate kinase 2	1.306
ZNF684	zinc finger protein 684	1.31

CCDC15	coiled-coil domain containing 15	1.313
DISC1	DISC1 scaffold protein	1.331
DAD1	defender against cell death 1	1.333
TCF7L1	transcription factor 7 like 1	1.333
UPK2	uroplakin 2	1.343
ZNF567	zinc finger protein 567	1.353
PDCD2L	programmed cell death 2 like	1.357
SYNGR3	synaptogyrin 3	1.359
PIGP	phosphatidylinositol glycan anchor biosynthesis class P	1.383
ARL14EP	ARF like GTPase 14 effector protein	1.388
GTPBP10	GTP binding protein 10	1.397
LY6S-AS1	LY6S antisense RNA 1	1.428
FANCI	FA complementation group I	1.449
UBE2Q2	ubiquitin conjugating enzyme E2 Q2	1.452
ZNF596	zinc finger protein 596	1.47
OCIAD1	OCIA domain containing 1	1.576
YPEL4	yippee like 4	1.663
ARHGEF10L	Rho guanine nucleotide exchange factor 10 like	1.669
ZNF552	zinc finger protein 552	1.689
ASPG	asparaginase	1.728
PLSCR1	phospholipid scramblase 1	1.784
SNCG	synuclein gamma	2.027
